# Effect of Vitamin D Supplementation on Outcomes in People With Early Psychosis: The DFEND Randomized Clinical Trial

**DOI:** 10.1001/jamanetworkopen.2021.40858

**Published:** 2021-12-28

**Authors:** Fiona Gaughran, Dominic Stringer, Gabriella Wojewodka, Sabine Landau, Shubulade Smith, Poonam Gardner-Sood, David Taylor, Harriet Jordan, Eromona Whiskey, Amir Krivoy, Simone Ciufolini, Brendon Stubbs, Cecilia Casetta, Julie Williams, Susan Moore, Lauren Allen, Shanaya Rathod, Andrew Boardman, Rehab Khalifa, Mudasir Firdosi, Philip McGuire, Michael Berk, John McGrath

**Affiliations:** 1 Department of Psychosis Studies, Institute of Psychiatry, Psychology, and Neuroscience, King’s College London, London, UK; 2 South London and Maudsley National Health Service (NHS) Foundation Trust, London, UK; 3 Department of Biostatistics and Health Informatics, Institute of Psychiatry, Psychology, and Neuroscience, King’s College London, London, UK; 4 King’s Clinical Trials Unit, King’s College London, London, UK; 5 Department of Forensic and Neurodevelopmental Science, Institute of Psychiatry, Psychology, and Neuroscience, King’s College London, London, UK; 6 Department of Psychological Medicine, Institute of Psychiatry, Psychology, and Neuroscience, King’s College London, London, UK; 7 Department of Health Service and Population Research, Institute of Psychiatry, Psychology, and Neuroscience, King’s College London, London, UK; 8 Department of Psychiatry, St Vincent’s University Hospital, Dublin, Ireland; 9 Department of Psychiatry, Royal College of Surgeons, Dublin, Ireland; 10 Clinical Trials Facility, Research Department, Tom Rudd Unit, Moorgreen Hospital, Southampton, UK; 11 Cheshire & Wirral Partnership NHS Trust, Chester, UK; 12 Kent and Medway NHS & Social Care Partnership Trust, NHS Trust, London, UK; 13 South West London and St George’s Mental Health NHS Trust, Queen Mary’s Hospital, London, UK; 14 Institute for Mental and Physical Health and Clinical Translation, Deakin University, School of Medicine, Barwon Health, Geelong, Australia; 15 Queensland Centre for Mental Health Research, The Park Centre for Mental Health, Wacol, Queensland, Australia; 16 Queensland Brain Institute, University of Queensland, Brisbane, Queensland, Australia; 17 National Centre for Register-Based Research, Aarhus University, Aarhus, Denmark

## Abstract

**Importance:**

People with psychotic disorders have an increased risk of vitamin D deficiency, which is evident during first-episode psychosis (FEP) and associated with unfavorable mental and physical health outcomes.

**Objective:**

To examine whether vitamin D supplementation contributes to improved clinical outcomes in FEP.

**Design, Setting, and Participants:**

This multisite, double-blind, placebo-controlled, parallel-group randomized clinical trial from the UK examined adults 18 to 65 years of age within 3 years of a first presentation with a functional psychotic disorder who had no contraindication to vitamin D supplementation. A total of 2136 patients were assessed for eligibility, 835 were approached, 686 declined participation or were excluded, 149 were randomized, and 104 were followed up at 6 months. The study recruited participants from January 19, 2016, to June 14, 2019, with the final follow-up (after the last dose) completed on December 20, 2019.

**Interventions:**

Monthly augmentation with 120 000 IU of cholecalciferol or placebo.

**Main Outcomes and Measures:**

The primary outcome measure was total Positive and Negative Syndrome Scale (PANSS) score at 6 months. Secondary outcomes included total PANSS score at 3 months; PANSS positive, negative, and general psychopathology subscale scores at 3 and 6 months; Global Assessment of Function scores (for symptoms and disability); Calgary Depression Scale score, waist circumference, body mass index, and glycated hemoglobin, total cholesterol, C-reactive protein, and vitamin D concentrations at 6 months; and a planned sensitivity analysis in those with insufficient vitamin D levels at baseline.

**Results:**

A total of 149 participants (mean [SD] age, 28.1 (8.5) years; 89 [59.7%] male; 65 [43.6%] Black or of other minoritized racial and ethnic group; 84 [56.4%] White [British, Irish, or of other White ethnicity]) were randomized. No differences were observed in the intention-to-treat analysis in the primary outcome, total PANSS score at 6 months (mean difference, 3.57; 95% CI, −1.11 to 8.25; *P* = .13), or the secondary outcomes at 3 and 6 months (PANSS positive subscore: mean difference, −0.98; 95% CI, −2.23 to 0.27 at 3 months; mean difference, 0.68; 95% CI, −0.69 to 1.99 at 6 months; PANSS negative subscore: mean difference, 0.68; 95% CI, −1.39 to 2.76 at 3 months; mean difference, 1.56; 95% CI, −0.31 to 3.44 at 6 months; and general psychopathology subscore: mean difference, −2.09; 95% CI, −4.36 to 0.18 at 3 months; mean difference, 1.31; 95% CI, −1.42 to 4.05 at 6 months). There also were no significant differences in the Global Assessment of Function symptom score (mean difference, 0.02; 95% CI, −4.60 to 4.94); Global Assessment of Function disability score (mean difference, −0.01; 95% CI, −5.25 to 5.23), or Calgary Depression Scale score (mean difference, −0.39; 95% CI, −2.05 to 1.26) at 6 months. Vitamin D levels were very low in the study group, especially in Black participants and those who identified as another minoritized racial and ethnic group, 57 of 61 (93.4%) of whom had insufficient vitamin D. The treatment was safe and led to a significant increase in 25-hydroxyvitamin D concentrations.

**Conclusions and Relevance:**

In this randomized clinical trial, no association was found between vitamin D supplementation and mental health or metabolic outcomes at 6 months. Because so few patients with FEP were vitamin D replete, the results of this study suggest that this group would benefit from active consideration in future population health strategies.

**Trial Registration:**

isrctn.org Identifier: ISRCTN12424842

## Key Points

**Question:**

Does monthly supplementation with 120 000 IU of vitamin D improve outcomes in people with early psychosis?

**Findings:**

This randomized clinical trial of 149 adults diagnosed with early psychosis found no evidence that vitamin D supplementation improved mental or physical health outcomes during a 6-month follow-up period. Vitamin D levels were very low, especially in Black participants and those who identified as other minoritized racial and ethnic groups, 93.4% of whom had insufficient levels.

**Meaning:**

These results suggest that although vitamin D did not improve 6-month mental or physical health outcomes in this population, public health strategies should take into account the very high prevalence of vitamin D deficiency and insufficiency, even in the early years of psychosis, when developing population-wide interventions.

## Teaser

This randomized clinical trial examines the effects of vitamin D supplementation in the treatment of early psychosis on mental health outcomes.

## Introduction

Vitamin D deficiency is more common in people with psychosis than in the general population.^1,2,3,4^ The concentration of 25-hydroxyvitamin D, the transport and storage form of vitamin D commonly used to assess overall vitamin D status, is low in many chronic mental and general medical conditions, including psychosis.^5,6^ This finding is thought to result from poor general health associated with sedentary lifestyles, less sun exposure, and poor general nutrition.^7^

Animal experiments have linked low vitamin D with changes in brain-related outcomes,^3,8,9^ triggering speculation that vitamin D supplementation may improve such outcomes^10^ and that vitamin D may be neuroprotective.^11^ The active form of vitamin D (1,25-dihydroxyvitamin D) can protect rodent brains from excitotoxic insults via L-type calcium channels,^12^ which it activates.^13^ Variants in the *CACNA1C* gene (OMIM 114205), which encodes an L-type calcium channel subunit, confer an increased risk of schizophrenia.^14,15^

On the basis of these^3,8,9,10,11,12,13,14,15^and other animal studies,^16,17^ it has been proposed that optimizing vitamin D status may improve outcomes in those with brain disorders.^18^ Although mendelian randomization does not suggest that vitamin D deficiency during adulthood increases the risk of developing brain dysfunction, it is feasible that it may impede recovery. Furthermore, a randomized clinical trial^19^ (RCT) in Parkinson disease found that those receiving vitamin D supplementation did not show disease progression seen in the placebo group.

To date, 1 RCT^20^ has examined vitamin D supplementation in patients with psychosis, with 14 000 IU weekly for 8 weeks showing no benefit over placebo in people with treatment-resistant schizophrenia. No studies have examined vitamin D augmentation in first-episode psychosis (FEP), a group with high rates of vitamin D deficiency (42.0%) and insufficiency (37.5%)^21^ and who may be more responsive to supplementation than those with established psychosis.^22,23,24^ Notably, higher vitamin D levels at first presentation were associated with fewer total and negative symptoms of psychosis a year later.^21^

We report the results of the DFEND (Vitamin D Supplementation Compared to Placebo in People With First Episode Psychosis–Neuroprotection Design) trial. We hypothesized that, compared with placebo, patients with FEP receiving vitamin D supplementation would have a greater reduction in Positive and Negative Syndrome Scale (PANSS) scores over time, with the PANSS subscores of global function and depression selected as secondary outcomes.^25^ Finally, reflecting evidence of the association between neuroprogression and somatoprogression^26^ and the associations among vitamin D deficiency, medical conditions, and mental disorders (eg, obesity, diabetes, and hypercholesterolemia),^32,33^ we included a range of biomarkers as secondary outcomes.

## Methods

The study was a double-blind, placebo-controlled RCT with participants allocated 1:1 into 2 treatment arms. Of 2136 potentially suitable participants, 166 provided written informed consent, of whom 149 were randomized (Figure). All data were deidentified. The full protocol for the trial has been previously published.^25^ The protocol (Supplement 1) and related documents were approved by the National Research Ethics Committee–London Dulwich, the Health Research Authority, and the Medicines and Healthcare Products Regulatory Agency for Clinical Trial Authorization and registered online. (Also see the eMethods in Supplement 2.) This study followed the Consolidated Standards of Reporting Trials (CONSORT) reporting guideline.^27^

**Figure.**
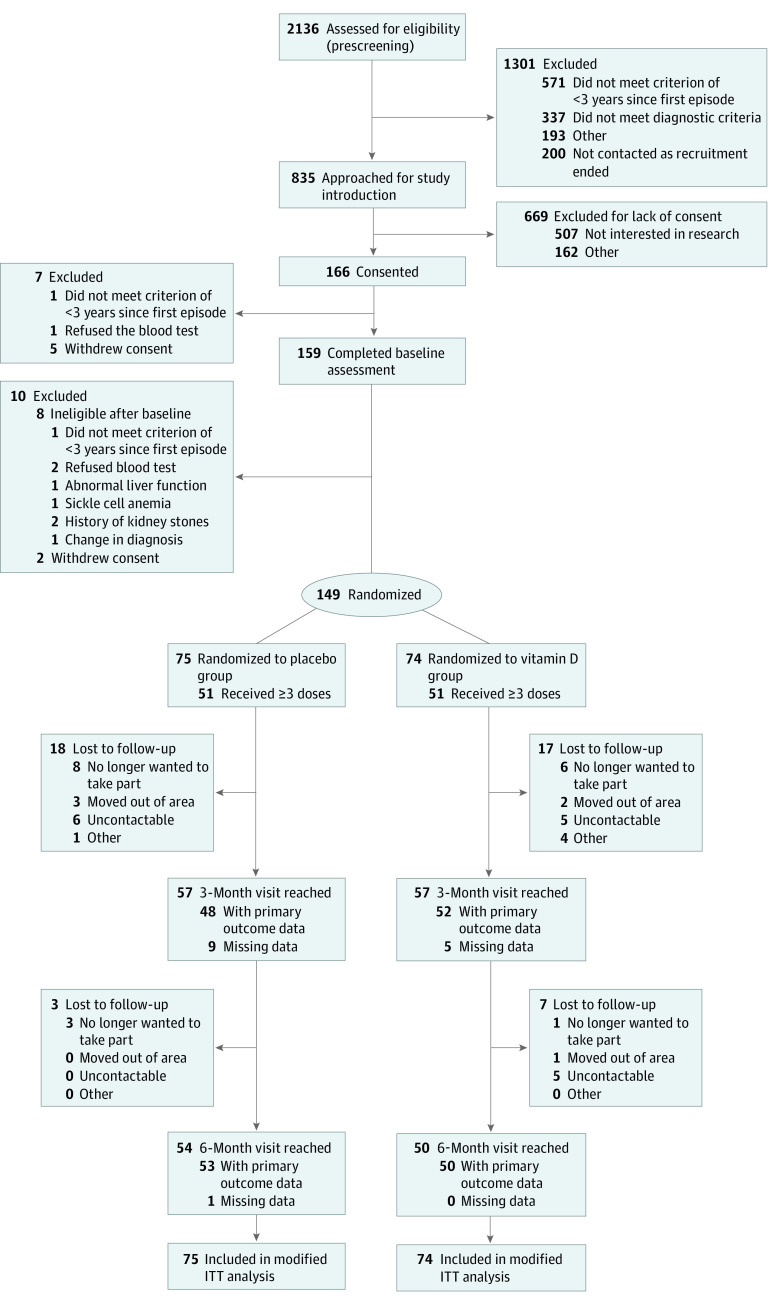
CONSORT Flow Diagram ITT indicates intention to treat.

Outcome measures were assessed at baseline with the primary outcome at month 6. Safety measures and PANSS scores^28,29^ were measured at month 3. To monitor adverse events, participants remained in the trial from consent to 28 days after their last dose.

After trial commencement, we adjusted several study features to optimize recruitment and retention, including (1) discontinuation of 12-month postrandomization follow-up, (2) increase of age range upper limit from 45 to 65 years (February 6, 2017), and (3) extension of the criterion stipulating FEP from the past 6 months to 3 years (July 8, 2016). During the trial, Public Health England recommended universal winter supplementation of 400 IU of vitamin D daily, so after safety checks, participants were permitted to take this dose alongside the study medication (February 12, 2016).

The study recruited participants from January 19, 2016, to June 14, 2019, with the final post–last dose follow-up completed on December 20, 2019. The study was performed at 5 sites in England: South London and Maudsley National Health Service (NHS) Foundation Trust, Southern Health NHS Foundation Trust, Cheshire and Wirral Partnership NHS Foundation Trust, Kent and Medway NHS and Social Care Partnership Trust, and South West London and St George’s Mental Health NHS Trust.

Inclusion criteria were age of 18 to 65 years, an *International Statistical Classification of Diseases and Related Health Problems, Tenth Revision (ICD-10)* diagnosis of functional psychosis (F20-F29, F30-33), agreement to refrain from multivitamin or nonstudy vitamin D supplements that exceeded 400 IU/d, willingness to give a baseline blood vitamin D sample, and written informed consent. Exclusion criteria were known intolerance to vitamin D_2_ or D_3_ or known allergy to any trial medication; currently taking vitamin D supplements that exceeded 400 IU/d; use of cardiac glycosides, calcium channel blockers, corticosteroids, bendroflumethiazide, isoniazid, or rifampicin in the past month; known active tuberculosis, sarcoidosis, hypoparathyroidism or hyperparathyroidism, past or present nephrolithiasis, suspected or diagnosed hepatic or renal dysfunction, malignant cancer (other than nonmelanoma skin cancer) not in remission for 3 years or more, or calcium disorders; baseline corrected serum calcium level greater than 10.4 mg/dL (to convert to millimoles per liter, multiply by 0.25); known history of hypercalcemia; being pregnant, breastfeeding, or planning pregnancy; lacking capacity to provide written informed consent; and insufficient English to complete core assessments with available assistance. Eligibility was confirmed by a physician trained in the DFEND study (F.G., S.S., A.K., S.C., C.C., S.M., S.R., A.B., R.K., and M.F.).

The investigational medicinal product was a 1-mL solution of 0.5 mg of cholecalciferol, equivalent to 20 000 IU of vitamin D_3_ (Vigantol oil, Merck GmbH). Placebo was an organoleptically matched triglyceride oil (Miglyol 812 oil; IOI Oleo GmbH). Both active and placebo products were packaged in identical glass bottles (volume of 8 mL). One dose was 6 mL of the active product, equivalent to 120 000 IU of vitamin D_3_, or 6 mL of placebo and was administered orally in a graduated syringe by a trained researcher (P.G.-S., H.J., S.C., B.S., C.C., J.W., and L.A.) monthly for 6 months, who visually assessed intervention adherence.

### Outcome Measures

Demographic data, including self-reported ethnicity and sex, were collected from participants. Outcome measures were completed at baseline and month 6, along with the PANSS score^28,29^ at month 3 (eTable 1 in Supplement 2). Blood samples were obtained at baseline, month 3, and month 6 for eligibility and safety checks. OPCRIT+ software was used by clinicians or trained researchers (A.K., S.C., C.C., J.W., S.R., A.B., R.K., and M.F.) on participant medical records to obtain standardized diagnoses.^30^

The primary outcome was total PANSS score at 6 months. The PANSS rates 30 symptoms on a 1- to 7-point scale, with higher scores indicating greater severity and a score of 58 indicating mild illness.^31^ Secondary outcomes were total PANSS score at 3 months; PANSS positive, negative, and general psychopathology subscores at 3 and 6 months; and the following measures at 6 months: Global Assessment of Function^35^; Calgary Depression Scale^36,37^; waist circumference; body mass index (calculated as weight in kilograms divided by height in meters squared); and glycated hemoglobin, total cholesterol, C-reactive protein, and vitamin D concentrations.

Participants were asked about any changes to their health, including symptoms of hypercalcemia, at all follow-up visits. Any serious adverse events, serious adverse reactions, or unexpected serious adverse reactions were reported by the research team within 24 hours to the chief investigator and the sponsor. Hospitalizations for deterioration in mental state were an anticipated serious adverse event, so excepted from 24-hour reporting. The data monitoring and ethics committee regularly reviewed all adverse and serious adverse events reported. Hospitalizations and contact with home treatment teams were recorded during the trial period. Adverse events and serious adverse events were reported by trial arm and summarized.

### Randomization

Participants were randomized 1:1 to treatment with vitamin D or placebo using randomly varying block sizes of 2 or 4 and stratified by ethnicity (2 levels: White and ethnicity other than White), as vitamin D levels vary depending on skin color.^38,39^ Randomization was via an online service through the King’s Clinical Trial Unit at King’s College London.

### Blinding

All participants and members of the research team were blinded throughout. At each monthly follow-up visit, participants were asked to guess if they were taking the investigational medical product or placebo. Trial statisticians were partially blinded (able to see data by arm without knowing which arm was placebo or vitamin D). Only pharmacists dispensing the study treatment and the study monitor had access to treatment allocation assigned to each specific participant identification number.

### Sample Size

The original target sample size was 240 people. For the power analyses, we modeled 2 plausible scenarios. For the primary outcome at 6 months, we assumed 20% attrition, with an effective sample size of 192 (96 in each trial arm). Based on α = .05 and power of 80%, samples between 200 and 180 participants would allow us to confidently detect mean PANSS total score group differences of 6 to 6.3 units, a standardized effect size of approximately 0.4 to 0.42.

Considering 90% power with the same assumptions, mean PANSS total score group differences of between 6.9 and 7.3 units (standard effect size of 0.46 to 0.49) could be detected. However, the study randomized only 149 participants, resulting in a reduction in power.

### Statistical Analysis

The primary analyses of efficacy used the intention-to-treat method (Supplement 1), using all available follow-up data from all randomized participants. The significance level was set at 2-sided *P* < .05 for all specified main and secondary analyses, with estimates and 95% CIs presented for all effects. The main objective of the formal statistical analyses was to assess the effect of vitamin D supplementation on the primary outcome (PANSS total score at 6-month follow-up). Originally, as per the statistical analysis plan, linear mixed modeling was to be used. Missingness was explored for the DFEND data set. Adherence to the intervention or placebo regimen was associated with outcome missingness in the primary outcome (χ^2^ = 73.66; *P* < .001). Because adherence is a posttreatment variable and therefore cannot be conditioned on, we used multiple imputation instead. Under a multiple imputation approach for missing data, there was no longer a substantial benefit to the more complex linear mixed-modeling approach; therefore, simpler linear regression models were used instead. The multiple imputation procedure provides valid inferences under a missing at random assumption whereby only observed variables, including nonadherence with the treatment, drive missingness (eAppendix in Supplement 2).

Regarding the multiple imputation procedure, briefly, for each outcome, the analysis model used was a linear regression with treatment arm, baseline outcome, and ethnicity (randomization stratifier) as explanatory variables. The imputation models contained all the variables of the analysis model(s) as well as factors associated with missingness: age (identified empirically to predict missingness, *P* = .03) and adherence (number of doses taken of either vitamin D or placebo, *P* < .001). Logistic regression was used with the same explanatory variables for the C-reactive protein level greater than 0.30 mg/dL (to convert to milligrams per liter, multiply by 10) outcome, and an odds ratio estimated.

Additional subgroup analyses were performed to examine the hypothesis that vitamin D supplementation may have been of greatest benefit in the subgroup with vitamin D deficiency or insufficiency (defined as <20 ng/mL of 25-hydroxyvitamin D [to convert to nanomoles per liter, multiply by 2.496]) at baseline.^40^ For these subgroup analyses, the analysis model was expanded to include binary baseline vitamin D status and the interaction product term as an extra explanatory variable, and the coding was chosen so the regression coefficients represented the treatment effect within the subpopulation of participants with deficient or insufficient vitamin D.

Sensitivity analyses were performed for the primary analysis to check the robustness of the results against departures from the missing at random assumption and to check the effect of changes to the inclusion and exclusion criteria. A mediation analysis was performed to test the hypothesis that vitamin D levels are a mediator of the effect of treatment on PANSS total score.

## Results

A total of 149 participants (mean [SD] age, 28.1 (8.5) years; 89 [59.7%] male; 65 [43.6%] Black or of another minoritized racial and ethnic group; 84 [56.4%] White [British, Irish, or of other White ethnicity]) were randomized. Demographic and clinical characteristics are given in Table 1 and Table 2. At baseline, the groups were comparable on all variables of interest. *ICD-10* diagnoses included schizophrenia, schizoaffective disorder (n = 53); affective disorders (n = 36); other nonorganic psychosis (n = 55); and no OPCRIT diagnosis (n = 5).

**Table 1.** Baseline Characteristics and Primary and Secondary Measures

Variable	Placebo		Vitamin D	
	No. of patients	Mean (SD)	No. of patients	Mean (SD)
Age, y	75	28.39 (8.39)	74	27.76 (8.74)
Baseline scores				
CDS	74	5.95 (5.24)	73	5.37 (5.26)
GAF				
Disability	75	62.59 (16.87)	74	62.28 (14.52)
Symptom	75	62.77 (16.27)	74	61.51 (14.51)
PANSS scores				
General psychopathology	75	29.60 (7.60)	74	28.97 (6.61)
Negative symptoms	75	12.56 (5.05)	74	12.72 (4.31)
Positive symptoms	75	15.12 (5.26)	74	14.81 (5.07)
Total	75	57.28 (14.27)	74	56.50 (12.38)
Secondary outcome measures				
BMI	75	26.44 (5.97)	73	25.94 (4.65)
Waist circumference, cm	71	90.57 (15.12)	69	91.55 (13.95)
Glycated hemoglobin, % (mmol/mol)	64	35.04 (4.66)	70	35.87 (4.60)
Total cholesterol, mg/dL	70	185.33 (43.24)	72	186.49 (48.65)
C-reactive protein, mg/dL	64	0.19 (0.20)	62	0.20 (0.33)
Vitamin D levels, ng/mL	71	15.93 (11.21)	71	14.30 (11.22)

Abbreviations: BMI, body mass index (calculated as weight in kilograms divided by height in meters squared); CDS, Calgary Depression Scale; GAF, Global Assessment of Function; PANSS, Positive and Negative Syndrome Scale.

SI conversion factors: To convert total cholesterol to millimoles per liter, multiply by 1.26; C-reactive protein to milligrams per liter, multiply by 10; glycated hemoglobin from percentage of total hemoglobin to proportion of total hemoglobin, multiply by 0.01; and vitamin D to nanomoles per liter, multiply by 2.496.

**Table 2.** Baseline Demographic Characteristics and Vitamin D Status

Characteristic	No. (%) of patients		
	Placebo	Vitamin D	Total
Sex			
Male	38 (50.7)	51 (68.9)	89 (59.7)
Female	37 (49.3)	23 (31.1)	60 (40.3)
Race and ethnicity			
White (British, Irish, or other White ethnicity)	42 (56.0)	42 (56.8)	84 (56.4)
Black or other minoritized racial and ethnic group	33 (44.0)	32 (43.2)	65 (43.6)
Vitamin D status			
≥20 ng/mL	21 (29.6)	15 (21.1)	36 (25.4)
<20 ng/mL	50 (70.4)	56 (78.9)	106 (74.6)

SI conversion factor: To convert vitamin D to nanomoles per liter, multiply by 2.496.

A total of 106 of the 142 participants (74.6%) had insufficient 25-hydroxyvitamin D concentrations (<20 ng/mL), with 58 (40.9%) frankly deficient (<10 ng/mL) (Table 2). Black race and other minoritized racial and ethnic group membership were associated with higher proportions of vitamin D insufficiency (57 of 61 [93.4%] vs 49 of 81 [60.5%]) and deficiency (33 of 61 [54.1%] vs 25 of 81 [30.9%]) compared with White race.

For the primary outcome (PANSS total score at 6 months), no group difference was found (mean difference, 3.57; 95% CI, −1.11 to 8.25; *P* = .13). In addition, there was no group difference at either time point in PANSS positive (mean difference, −0.98; 95% CI, −2.23 to 0.27 at 3 months; mean difference, 0.68; 95% CI, −0.69 to 1.99 at 6 months), negative (mean difference, 0.68; 95% CI, −1.39 to 2.76 at 3 months; mean difference, 1.56; 95% CI, −0.31 to 3.44 at 6 months), or general psychopathology (mean difference, −2.09; 95% CI, −4.36 to 0.18 at 3 months; mean difference, 1.31; 95% CI, −1.42 to 4.05 at 6 months) subscores. There were also no differences in Global Assessment of Function symptom (mean difference, 0.02; 95% CI, −4.60 to 4.94) or disability (mean difference, −0.01; 95% CI, −5.25 to 5.23) scores or Calgary Depression Scale score (mean difference, −0.39; 95% CI, −2.05 to 1.26) (Table 3 and Table 4). Body mass index (mean difference, 0.30; 95% CI, −0.63 to 1.23), waist circumference (mean difference, −0.72; 95% CI, −4.23 to 2.79), glycated hemoglobin level (mean difference, −0.75; 95% CI, −2.23 to 0.73), total cholesterol level (mean difference, −0.03; 95% CI, −0.45 to 0.39), and C-reactive protein level (odds ratio, 0.62; 95% CI, 0.17-2.21) likewise had no statistically significant group differences (Table 4). Those randomized to cholecalciferol had a large increase in 25-hydroxyvitamin D concentration compared with the placebo group (16.0 ng/mL; 95% CI, 11.10-11 839.80 ng/mL; *P* = 0.01).

**Table 3.** Efficacy Measures: Primary and Secondary Outcomes at 3 and 6 Months

Outcome measure	3 Months				6 Months			
	No. of patients	Mean (SD)	Mean difference (95% CI)	*P* value	No. of patients	Mean (SD)	Mean difference (95% CI)	*P* value
PANSS total score								
Placebo	48	53.99 (14.55)	1 [Reference]	NA	53	53.04 (14.16)	1 [Reference]	NA
Vitamin D	52	50.57 (14.65)	−2.43 (−6.98 to 2.12)	.29	50	55.88 (17.46)	3.57 (−1.11 to 8.25)	.13
PANSS positive symptoms score								
Placebo	48	13.80 (5.36)	1 [Reference]	NA	53	13.64 (5.05)	1 [Reference]	NA
Vitamin D	52	14.34 (6.77)	−0.98 (−2.23 to 0.27)	.12	50	14.84 (6.31)	0.68 (−0.69 to 1.99)	.34
PANSS negative symptoms score								
Placebo	48	11.48 (4.63)	1 [Reference]	NA	53	10.87 (4.07)	1 [Reference]	NA
Vitamin D	52	10.44 (3.66)	0.68 (−1.39 to 2.76)	.52	50	11.58 (5.19)	1.56 (−0.31 to 3.44)	.10
PANSS general psychopathology score								
Placebo	48	28.71 (7.86)	1 [Reference]	NA	53	28.53 (7.85)	1 [Reference]	NA
Vitamin D	52	25.79 (7.18)	−2.09 (−4.36 to 0.18)	.07	50	29.46 (9.55)	1.31 (−1.42 to 4.05)	.34

Abbreviations: NA, not applicable; PANSS, Positive and Negative Syndrome Scale.

**Table 4.** Efficacy Measures: Secondary Outcomes at 6 Months

Outcome measure	No. of patients	Mean (SD)	Mean difference (95% CI)	*P* value
GAF symptom score				
Placebo	53	66.98 (13.60)	1 [Reference]	NA
Vitamin D	50	68.14 (14.34)	0.02 (−4.60 to 4.94)	.99
GAF disability score				
Placebo	53	65.85 (15.81	1 [Reference]	NA
Vitamin D	50	67.62 (14.98)	−0.01 (−5.25 to 5.23)	.99
CDS score				
Placebo	53	5.40 (4.97)	1 [Reference]	NA
Vitamin D	50	4.44 (4.57)	−0.39 (−2.05 to 1.26)	.64
Waist circumference, cm				
Placebo	47	93.60 (17.01)	1 [Reference]	NA
Vitamin D	46	94.35 (17.11)	−0.72 (−4.23 to 2.79)	.68
BMI				
Placebo	50	27.32 (6.41)	1 [Reference]	NA
Vitamin D	47	26.68 (5.44)	0.30 (−0.63 to 1.23)	.52
Glycated hemoglobin, % (mmol/mol)				
Placebo	44	35.23 (5.88)	1 [Reference]	NA
Vitamin D	36	35.26 (4.59)	−0.75 (−2.23 to 0.73)	.31
Total cholesterol, mg/dL				
Placebo	49	186.87 (50.19)	1 [Reference]	NA
Vitamin D	43	183.40 (42.08)	−0.03 (−0.45 to 0.39)	.88
C-reactive protein >0.30 mg/dL, No. (%)^a^				
Placebo	41	1.10 (2.68)	1 [Reference]	NA
Vitamin D	34	0.80 (2.35)	0.62 (0.17 to 2.21)	.46
Vitamin D blood levels, ng/mL				
Placebo	50	15.89 (8.80)	1 [Reference]	NA
Vitamin D	42	32.97 (15.40)	39.98 (27.70 to 52.27)	<.001

Abbreviations: BMI, body mass index (calculated as weight in kilograms divided by height in meters squared); CDS, Calgary Depression Scale; GAF, Global Assessment of Function; NA, not applicable.

SI conversion factors: To convert total cholesterol to millimoles per liter, multiply by 1.26; C-reactive protein to milligrams per liter, multiply by 10; and vitamin D to nanomoles per liter, multiply by 2.496.

^a^ The distribution of the C-reactive protein was dichotomized as 0.30 mg/dL or less and greater than 0.30 mg/dL. For this outcome, an odds ratio is reported.

In the 106 participants (74.6%) with baseline 25-hydroxyvitamin D levels less than 20 ng/mL, results were similar to the overall results of no group differences in any outcome measures, with the mean difference in PANSS score between the vitamin D and placebo groups being 3.21 (95% CI, −2.21 to 8.63) at 6 months and −2.57 (95% CI, −7.93 to 2.79) at 3 months (eTables 2 and 3 in Supplement 2).

Sensitivity analyses found that results for the primary outcome were robust to changes in eligibility criteria and to departures from the missing-at-random assumption (eTable 4 and eFigure 1 in Supplement 2). Mediation analysis found no evidence that blood vitamin D levels mediated the association between trial arm and total PANSS scores (despite clear evidence of an effect of trial arm on blood vitamin D levels) (eFigure 2 in Supplement 2).

There was no evidence of any systemic participant unblinding. A χ^2^ test comparing participants who guessed they were taking vitamin D at 6 months (14 of 49 [28.6%] in the vitamin D arm and 15 of 53 [28.3%] in the placebo arm) against those who guessed placebo or answered do not know showed no difference (*P* = .98). Only 2 of 74 participants (2.7%) had a potential adverse drug reaction (vs 3 of 75 [4.0%] in the placebo arm) (eTables 12, 13, 14, 15, 16, and 17 in Supplement 2).

Hospitalization rates were 18.9% in the test and 25.3% in the control groups, with a mean (SD) length of stay if admitted of 33.5 (31.1) days in the test group and 27.8 (31.9) days in the control group (eTables 5, 6, 7, and 8 in Supplement 2). Of those randomized to vitamin D, 5 of 74 (6.8%) had contact with a home treatment team compared with 7 of 75 controls (9.3%). Of those with home treatment team input, the mean (SD) numbers of contacts were 15.4 (5.4) in the test group and 12.6 (6.3) in the control group (eTables 9, 10, and 11 in Supplement 2).

## Discussion

In this randomized clinical trial, we found no evidence to support the hypothesis that vitamin D supplementation leads to better mental health outcomes in those with FEP. In addition, we did not find benefit for cardiometabolic risk factors. The prevalence of vitamin D insufficiency and deficiency was high (74.6%); thus, the sample was well suited to detecting any potential benefits that may have arisen from correcting this. However, even in this subgroup, there was no evidence to support the guiding hypothesis.

The expectation for nutritional agents used as augmentation for psychosis is not that they would be effective treatments in isolation.^41,42^ Rather, given the suboptimal effectiveness and adverse effects of antipsychotics, the possibility that safe, cheap, and acceptable nutritional agents may provide small to moderate benefits is important to examine, although this was not evident in this study, despite an increase in vitamin D levels in the test group.

We included secondary cardiometabolic biomarker outcomes but found no group differences. Several well-powered community-based studies of the association of vitamin D supplements with a range of general health outcomes have been published recently,^32,41,43,44,45^ with the general consensus that the initial optimism about potential general health benefits of vitamin D supplementation is no longer supported.^42^

The findings of a previous mendelian randomization study^46^ are consistent with the lack of efficacy seen in RCTs. A recent genome-wide association study^7^ performed bidirectional mendelian randomization, finding correlations between the genetic variations associated with schizophrenia and 25-hydroxyvitamin D but no evidence to suggest that genetically determined 25-hydroxyvitamin D concentration increased the risk of mental disorders. However, there was evidence that several mental disorders, including schizophrenia, may cause vitamin D deficiency, most likely because of behavioral changes after onset. These genetically informed studies^7,46^ do not support the hypothesis that low vitamin D levels may increase the risk of schizophrenia but emphasize the need for clinicians to monitor for vitamin D deficiency in those with mental disorders and recommend supplements or outdoor activity to optimize bone health.

With respect to clinical practice, we cannot now recommend monthly treatments with 120 000 IU of cholecalciferol in FEP. However, we note with concern the high prevalence of low vitamin D, especially in the sample of Black individuals and those identifying as another minoritized racial and ethnic group, 93.4% of whom had insufficient concentrations. People with psychosis have lower bone mineral density than controls,^47^ aggravated by the adverse effect of antipsychotics on bone.^48^ Low vitamin D levels can contribute to this common and potentially disabling condition.

### Limitations

This study has some limitations. It is unlikely that that our finding is a reflection of lack of power because even the best improvement that would be considered plausible according to the 95% CI (lower limit of −1.11 points) was very limited. The relatively short 6-month duration of treatment in this study may be a factor. Well-accepted vitamin D–related health outcomes, such as osteoporosis, are long-latency disorders.^49^ However, there are trade-offs between trial retention and adherence. The regular bolus monthly dose used in this study had several advantages, including not adding to the regular medication load and allowing the team to administer the supplements and observe adherence. However, there is some evidence to suggest that bolus doses of cholecalciferol may not be as effective as daily doses for some health outcomes^50,51^ and may be associated with an increased risk of adverse events, such as falls.^52^ We found no increase in adverse events, but future studies may wish to examine the association of vitamin D with brain-related outcomes based on longer periods of treatment and administered as daily rather than bolus treatments.

## Conclusions

Despite considerable public interest in the association between vitamin D and diverse health outcomes, the results from RCTs have deflated these expectations.^42^ The findings of the current study similarly do not provide evidence that vitamin D supplementation for 6 months shows benefit in the treatment of FEP but highlight that only a few individuals in this group are vitamin D replete and thus may benefit from particular attention in any future public health strategies.

## Notes

Supplement 1.
Trial Protocol and Statistical Analysis Plan

Supplement 2. **eMethods.** Supplemental Methods **eTable 1.** Outcome Measures and Timeline of the DFEND Study **eAppendix.** Supplemental Results **eTable 2.** Subgroup Analysis for Efficacy Measures (PANSS), at 3- and 6-Months, With Insufficient Vitamin D at Baseline (<50 25-D Nmol/L, N = 106) **eTable 3.** Subgroup Analysis for Efficacy Measures (GAF, CDS, BMI, Waist Circumference, Biochemical), at 6-Months With Insufficient Vitamin D at Baseline (<50 25-D nmol/L, N = 106) **eTable 4.** Results From Sensitivity to Inclusion/Exclusion Criteria Analysis **eTable 5.** Number of Participants That Had Inpatient Stays by Trial Arm (All) **eTable 6.** Summary of Inpatient Days for Participants Who Had Inpatient Stays by Trial Arm (All) **eTable 7.** Number of Participants That Had Inpatient Stays by Trial Arm (Post-Randomisation Admissions Only) **eTable 8.** Summary of Inpatient Days for Participants Who Had Inpatient Stays by Trial Arm (Postrandomisation Admissions Only) **eTable 9.** Number of Participants That Had at Least 1 Home Treatment Contact by Trial Arm **eTable 10.** Summary of Home Treatment Contacts for Participants Who Had at Least 1 Home Treatment Contact by Trial Arm **eTable 11.** Summary of Home Treatment Contacts for All Participants by Trial Arm **eTable 12.** Adverse Events by Trial Arm **eTable 13.** Total Number of Adverse Events by Body System Code and Trial Arm **eTable 14.** Number of Adverse Drug Reactions by Trial Arm **eTable 15.** List of Adverse Drug Reactions **eTable 16.** Number of Serious Adverse Events by Trial Arm **eTable 17.** List of All SAEs **eFigure 1.** Sensitivity to Missing at Random Assumption **eFigure 2.** Mediation Diagram

Supplement 3.
Data Sharing Statement
