# Supplementary material for: Effect of Vitamin D Supplementation on Outcomes in People With Early Psychosis: The DFEND Randomized Clinical Trial
Source: JAMA Netw Open. 2021 Dec 28;4(12):e2140858. doi: 10.1001/jamanetworkopen.2021.40858 (PMC8715346; doi:10.1001/jamanetworkopen.2021.40858)
Supplement: Supplement 1. — Trial Protocol and Statistical Analysis Plan [file jamanetwopen-e2140858-s001.pdf]

PROTOCOL FULL TITLE: A randomised, double-blind, placebo-controlled, parallel-group trial of Vitamin D supplementation compared to placebo in people presenting with their First Episode of psychosis Neuroprotection Design (DFEND)

Protocol Short Title/Acronym: DFEND

Version 10.1 Date 03.05.2019

Trial Identifiers

EudraCT Number	2014-002639-32
REC Number	14/LO/1588
Protocol number	3310
IRAS	147978

Co-Sponsors

King's College London, and South London and Maudsley NHS Foundation Trust
Contact: Helen Critchley, King's Health Partners Clinical Trials Office
F16 Guy's Tower, Guy's Hospital, Great Maze Pond, London SE1 9RT
Phone: 020 7188 5732 Fax: 020 7188 8330
Email: helen.critchley @kcl.ac.uk

Chief Investigator
Name: Dr Fiona Gaughran
Address: South London & Maudsley NHS Trust,
National Psychosis Unit, Royal Bethlem Hospital,
Monks Orchard Rd,
Beckenham, BR3 3BX
Telephone: 07794594220
Fax: *0203 2884312*
Email: fiona.p.gaughran@kcl.ac.uk

Name and address of Co-Investigator(s), Statistician, Laboratories etc.

Name: Professor John McGrath
Address: Queensland Brain Institute and Queensland Centre for Mental Health Research, QBI Building 79 Room: 534 Brisbane, QLD4072
Telephone: +61 7 334 66372
Fax: +61 7 3271 8698
Email: j.mcgrath@uq.edu.au

Name: Dr Shubulade Smith
Address: South London and Maudsley NHS Foundation Trust and Institute of Psychiatry, Kings College London. Department of Forensic and Neurodevelopmental Studies, PO Box 23 De Crespigny Park, London SE5 8AF
Telephone: +44 (0)20 7848 0694
Email: shubulade.smith@kcl.ac.uk

Name: Dr Michael Berk
Address: Deakin University Australia, Psychiatric Health Strategic Research Centre, School of Medicine Deakin University, Geelong Waurn Ponds Campus, VIC3220
Telephone: +61 3 4215 3320
Email: MIKEBE@BarwonHealth.org.au

Name: Professor David Taylor
Address: Kings College London and South London and Maudsley NHS Foundation Trust, Institute of Pharmaceutical Science King's College London 5th Floor, Franklin-Wilkins Building, 150 Stamford Street, London SE1 9NH
Telephone: 020 3228 5040
Email: david.taylor@slam.nhs.uk

Name: Professor Philip McGuire
Address: Department of Psychosis Studies Institute of Psychiatry De Crespigny Park, London SE5 8AF
Telephone: +44 (0) 207 848 0355
Email: philip.mcguire@kcl.ac.uk

Name: Professor Sabine Landau
Address: Room S206 Institute of Psychiatry De Crespigny Park, London SE5 8AF
Telephone: + 44 (0)20 7848 0313
Email: sabine.landau@kcl.ac.uk

Name: Professor Sir Robin Murray
Address: Department of Psychosis Studies Institute of Psychiatry De Crespigny Park, London SE5 8AF
Telephone: + 44 (0)2078480100/145
Fax: +44 (0)2078480287
Email: robin.murray@kcl.ac.uk

Table of Contents

1. Study Synopsis 5
2. Glossary of Terms........ 8
3. Background & Rationale 9
4. Trial Objectives and Design........12
4.1. Trial Objectives........ 12
4.2 Trial Endpoints 12
4.3 Trial Design........ 13
4.4 Trial Flowchart 14
5. Trial Medication 15
5.1 Investigational Medicinal Product 15
5.2 Dosing Regimen 15
5.3 IMP Risks........ 15
5.4 Drug Accountability 17
5.5 Storage of IMP 18
5.6 Subject Compliance 18
5.7 Concomitant Medication 19
6. Selection and Withdrawal of Subjects........19
6.1 Inclusion Criteria 19
6.2 Exclusion Criteria........ 19
6.3 Selection of Participants and informed consent 20
6.4 Randomisation Procedure / Code Break 20
6.4.1 Randomisation 20
6.4.2 Emergency Code Break........21
6.5 Withdrawal of Subjects 21
6.6 Expected Duration of Trial 22
7. Trial Procedures 22
7.1 By Visit........ 22
7.2 Laboratory Tests........ 25
8. Assessment of Efficacy 26
8.1 Efficacy Parameters 26
8.1.1 Primary Efficacy Parameters........26
8.1.2 Secondary Efficacy Parameters........26
8.1.3 Exploratory moderator analysis 26
8.2 Procedures for Assessing Efficacy Parameters........ 27
9. Assessment of Safety 27
9.1 Specification, Timing and Recording of Safety Parameters........ 27

9.2 Procedures for Recording and Reporting Adverse Events 28
9.2.1 Adverse events that do not require reporting 29
9.3 Treatment Stopping Rules 30
10. Statistics...30
10.1 Sample Size.. 30
10.2 Analysis 31
11. Trial Steering Committee...32
12. Data Monitoring Committee...32
13. Direct Access to Source Data and Documents...33
14. Ethics & Regulatory Approvals..34
15. Quality Assurance 34
16. Data Handling 34
17. Data Management 35
18. Publication Policy 36
19. Insurance / Indemnity 36
20. Financial Aspects..37
21. Signatures 37
22. DFEND protocol reference list: 38

1. Study Synopsis

Title of clinical trial	A randomised, double-blind, placebo-controlled, parallel-group trial of Vitamin D supplementation compared to placebo in people presenting with their First Episode of psychosis Neuroprotection Design (DFEND)
Protocol Short Title/Acronym	DFEND
Trial Phase if not mentioned in title	Double-blind, placebo-controlled, parallel-group trial of Vitamin D supplementation compared to placebo
Sponsor name	King's College London and South London and Maudsley NHS Foundation Trust
Chief Investigator	Dr Fiona Gaughran
Eudract number	2014-002639-32
REC number	14/LO/1588
Medical condition or disease under investigation	First Episode Psychosis
Purpose of clinical trial	We hypothesise that optimal vitamin D status could be neuroprotective in those with first episode psychosis (FEP).
Primary objective	To determine whether the addition of 120,000 IU monthly of vitamin D (cholecalciferol) supplement to standard treatments is more efficacious than placebo in improving outcomes (Positive And Negative Syndrome Scale Total score – PANSS) at 6 month follow-up in those with First Episode Psychosis.
Secondary objective (s)	To examine PANSS total score and related subscores (Positive Scale, Negative Scale and General Psychopathology Scale) at 3 and 6 months, and a broader range of clinically-relevant outcomes based on Global Assessment of Function (GAF), the Calgary Depression Scale, and 25OHD vitamin D concentrations, along with cardiovascular risk markers (Waist circumference, BMI HBA1c, Total Cholesterol & CRP).<br>We will examine these outcomes in (a) all participants

	regardless of baseline vitamin D status and (b) including a subgroup of patients with suboptimal vitamin D concentrations at baseline.
Tertiary Objective	To collect data on inflammatory/immune markers
Trial Design	Double-blind, placebo-controlled, parallel-group trial of Vitamin D supplementation compared to placebo
Endpoints	Primary: PANSS score at 6 month follow-up<br><br>Secondary: PANSS total score and related subscores (Positive Scale, Negative Scale and General Psychopathology Scale) at 3 and 6 months, clinically-relevant outcomes based on Global Assessment of Function (GAF), the Calgary Depression Scale, and 25OHD vitamin D concentrations, along with cardiovascular risk markers (Waist circumference, BMI, HBA1c, Total Cholesterol & CRP).
Sample Size	240 participants
Summary of eligibility criteria	All patients within Psychosis Services, including Early Intervention Services (EIS) and First Episode Psychosis (FEP) inpatient units who meet the following criteria will be invited to participate in the study:<br>• Aged between 18- 65 years old including women of child-bearing age.<br>• Having a first diagnosis of functional psychosis – FEP defined according to ICD-10 criteria for psychosis (codes F20-29 and F30-33)<br>• Willing to refrain from taking multivitamin or non-study vitamin D supplements, including cod liver oil, that exceed 400IU/day of vitamin D throughout the study.
IMP, dosage and route of administration	50% of participants will be randomised to Active Treatment with Cholecalciferol (vitamin D3), 120,000 IU per month (equivalent to 4,000 IU per day), given orally as Vigantol® oil by Merck GmbH (cholecalciferol dispersed in triglyceride oil as vehicle; 8 ml bottles, containing 20,000 IU cholecalciferol per 1ml drop, and administered as 6mL given in a graduated oral syringe by a fully trained member of the research team).

Active comparator product(s)	For the 50% receiving placebo, we will use identical bottles containing an organoleptically matched triglyceride oil (Miglyol® 812 oil).
Maximum duration of treatment of a Subject	6 months
Version and date of protocol amendments	V1, dated 07.08.2014<br>V2, dated 28.05.2015<br>V3, dated 24.08.2015<br>V4, dated 08.04.2016<br>V5, dated 11.05.2016<br>V6, dated 02.12.2016<br>V7, dated 02.06.2017<br>V8, dated 23.08.2017<br>V9, dated 11.04.2018 (retracted, not in use)<br>V9.1, dated 06.06.2018<br>V9.2, dated 01.10.2018<br>V10, dated 06.02.2018 (not in use)

2. Glossary of Terms

EIS	Early Intervention Services
FEP	First Episode Psychosis
OPCRIT	OPerational CRITeria
TMF	Trial Master File
ICH-GCP	International Conference on Harmonisation-Good Clinical Practice
GAF	Global Assessment of Function
PANSS	Positive And Negative Syndrome Scale
TSC	Trial Steering Committee
DMC	Data Monitoring Committee

3. Background & Rationale

The links between vitamin D status and brain function have strengthened considerably in the last decade (Deluca et al., 2013). Convergent evidence from epidemiology, basic neuroscience, experimental animal models and clinical trials now connects low vitamin D status with an increased risk of a wide range of neurological and psychiatric disorders.

Vitamin D belongs to a group of fat-soluble secosteroids. Ultraviolet B radiation on the epidermis converts a cholesterol metabolite to vitamin D3 (cholecalciferol; a preprohormone). This is subsequently hydroxylated to 25-hydroxyvitamin D3 (25OHD), a prohormone commonly used to measure vitamin D status. A second hydroxylation of this molecule converts 25OHD to the active secosteroid hormone, 25-dihydroxyvitamin D3 (1,25OHD). This hormone binds the vitamin D receptor (VDR), a member of the nuclear receptor superfamily, which includes receptors for other ligands known to be important in brain development and adult brain function (e.g. retinoic acid, thyroid hormone). The brain distribution of the VDR, and the enzyme required for the final hydroxylation have been mapped (Eyles et al., 2005). In the adult brain the VDR is most strongly expressed in large tyrosine hydroxylase positive (dopaminergic) neurons and this is of particular relevance to the current protocol, (Cui et al., 2013b). In addition, there is in vitro evidence that 1,25 dihydroxyvitamin D increases the expression of tyrosine hydroxylase (Puchacz et al., 1996).

There is robust and consistent evidence based on rodent experiments that developmental vitamin D deficiency alters a wide range of adult brain outcomes (Eyles et al., 2013). More recently, it has also been demonstrated that vitamin D deficiency of only 2-3 months duration in adult mice leads to alterations in behaviour, brain neurochemistry and GAD65/67 levels (Groves et al., 2013). Curiously, recent evidence from GWAS studies related to both schizophrenia and bipolar disorder have implicated common variants in genes coding for L-type calcium channel subunits (e.g. CACNA1C). The expression of this gene (and protein) are strongly linked to vitamin D concentration (Brewer et al., 2001).

Vitamin D and schizophrenia - evidence from epidemiology
The field has long been aware that schizophrenia is more common in those born in winter and spring (Torrey et al., 1997, Davies et al., 2003;). The prevalence of schizophrenia is associated with latitude – a finding first described by Fuller Torrey over 25 years ago (Torrey, 1987) and since replicated in systematic reviews (Davies et al., 2003). These findings, and the evidence that dark-skinned minority groups in cold countries have an increased risk of schizophrenia (Cantor-Graae and Selten, 2005) has led to the hypothesis that low vitamin D (especially during early life) may be implicated in schizophrenia (McGrath et al., 2010a). A study based on Danish neonatal dried blood spots has supported this hypothesis (McGrath et al., 2010b). Concerning the impact of low vitamin D on the adolescent and adult brain, a recent study based on a UK birth cohort (n = 3,182) found an association between low vitamin D during childhood (mean age 9 years) and an increased risk of psychotic symptoms during adolescence (mean age 14 years)(Tolppanen et al., 2012). A large population-based study of Swedish women (n = 33,623) found a significantly increased risk of psychotic-like experiences in those with low vitamin D intake (Hedelin et al.,

2010). Thus, the evidence suggests that low vitamin D not only disrupts early brain development but may also compromise later periods of brain growth and maturation.

Optimal vitamin D concentration is neuroprotective

There is robust evidence from in vitro and animal experiments indicating that vitamin D is neuroprotective (McCann and Ames, 2008). For example, vitamin D is a potent inducer of nerve growth factor (NGF) (Feron et al., 2005). In addition, vitamin D has a direct neuroprotective action against excitotoxic insults by down regulating L-type calcium channels (Brewer et al., 2001) and pre-treatment with vitamin D attenuates the effects of various stressors, including 6-hydroxydopamine-induced neurotoxicity (McCann and Ames, 2008). An experimental study based on a rodent ischemic stroke model reported that animals allocated to a vitamin D deficient diet (prior to the stroke lesion) subsequently had significantly greater ischemic brain damage and worse functional impairments (compared to rodents on vitamin D replete chow) (Balden et al., 2012). Furthermore, Suzuki and colleagues recently reported the outcomes of a double-blind, placebo-controlled trial of vitamin D supplementation (1,200 IU per day, for one year) on various Parkinson's Disease (PD) related outcomes(Suzuki et al., 2013). There were clear group differences on several of the outcomes. Those on placebo (and thus, those more likely to have persisting vitamin D insufficiency or deficiency), had a steady worsening on PD outcomes. In contrast, those on vitamin D supplements had no change in PD outcomes over the year. The results strongly suggest that low vitamin D status exacerbates disease progression (Cui et al., 2013a).

Could low vitamin D impair recovery from first episode psychosis?

Previously, we proposed that first generation dark-skinned migrants exposed to both ''social defeat'' and hypovitaminosis D may be less able to buffer stress-related adverse brain outcomes (McGrath, 2010). In light of the neuroprotective properties of vitamin D (especially those related to dopaminergic pathways), and in light of the recent findings that vitamin D supplementation can slow the progression of dopamine-related neurodegenerative disorders like PD, we believe that there is now a strong case to examine whether vitamin D supplementation can optimize outcomes in first episode psychosis (FEP) (McGrath, 2010).

A recently published systematic review (based on seven studies) has confirmed that those with psychosis are significantly more likely to have low concentrations of vitamin D (BelvederiMurri et al., 2013). Crews et al., 2013 recently examined vitamin D status in a sample of 69 patients with FEP and 69 controls matched on age, sex and ethnicity. FEP patients had nearly a three-fold increased risk of vitamin D deficiency (OR= 2.99, 95% CI=1.33-6.74). In the FEP group, over a third (36.2%) had frank vitamin D deficiency (less than 25 nmol/L) while an additional 25% of FEP were classified as having vitamin D insufficiency (between 25 and 50 nmol/L). As pilot data for this application, we undertook a preliminary analysis of data currently being collected for the IMPACT study (www.kcl.ac.uk/iop/depts/ps/research/clinicaltrials/IMPACT.aspx). Low vitamin D at baseline predicted poorer function (r=.327, p=0.045) and higher Calgary depression scores (r=.404, p=0.012) at 12 months. A significant relationship between baseline Vitamin D and 12 months PANSS did not persist once adjusted or age, sex, ethnicity and season of sampling, but mean vitamin D between baseline and 3 months tended towards an association with PANSS

total (r= -.356, p=0.063) and negative (r= -.342, p=0.059) scores at 12 months, and had a significant relationship with 12 month Depression (r=-.369; p=0.038) and GAF (r=.491, p=0.002).

Graham et al (2014) reported that in psychosis, greater severity of negative symptoms was correlated with lower vitamin D status (r = −0.55, P = 0.012), as were more severe overall cognitive deficits (r = 0.56, P = 0.019). Even though the numbers were modest, the correlations of overall symptom severity and positive symptom severity with 25OHvitamin D levels approached significance(r = −0.42, P = 0.07 and r =−0.36, P = 0.12, respectively).

We have identified several open-label, non-controlled studies that have used vitamin D supplements in those with psychotic disorders (Tiangga and Gowda, 2008, Dealberto, 2013; Thakurathi et al., 2013;), but these have focused on general health outcomes (e.g. metabolic syndrome) and 25OHD concentrations. We are aware of one RCT currently underway in Israel (Geha Mental Health Center; NCT01759485) which aims to explore if vitamin D supplements improve outcomes in clozapine-treated patients with chronic schizophrenia. To the best of our knowledge, there have been no trials examining vitamin D supplementation in FEP.

Could vitamin D supplementation improve recovery from FEP?
Mindful of a recent study suggesting that fish oil supplement can reduce the progression of high-risk individuals to psychosis (Amminger et al., 2010), we speculate that optimal vitamin D status could be neuroprotective in those with FEP. In particular, we believe that in FEP patients, suboptimal vitamin D will be associated with worse outcomes compared to those with normal vitamin D concentrations (e.g. at least 50 nmol/L). Thus, we hypothesize that vitamin D supplements will be associated with better outcomes.

Apart from the core symptoms associated with FEP, there is evidence that persistent low vitamin D may also compound the poor health status that is already associated with psychotic disorders (Leucht et al., 2007, Berk et al., 2008). For a variety of reasons related to shared risk biomarkers, lifestyle and the side-effects of medication, it is widely accepted that people with psychotic disorders have an increased risk of osteoporosis (Kishimoto et al., 2012, Partti et al., 2010, Meaney and O'Keane, 2007). In addition, there is evidence from observational epidemiology linking low vitamin D with an increased risk of (a) metabolic syndrome (Hypponen et al., 2008), (b) adverse cardiovascular outcomes (Melamed et al., 2008) and (c) some types of cancers (Plum and DeLuca, 2010). Large population-based, placebo-controlled studies of vitamin D supplementation are currently underway that will directly examine these research questions (Kupferschmidt, 2012).

There continues to be clinical equipoise regarding the benefits and cost-effectiveness of routine testing and supplementation of vitamin D in people with psychosis. A prescribing protocol raising awareness of the risk of vitamin D insufficiency and suggesting maintenance and supplementation strategies has recently been introduced to the local psychosis service by the London-based investigators on this protocol. Routine testing in the absence of symptoms of deficiency is not suggested, in view of the high cost of testing and the low toxicity of supplements (see appendix). Even if optimal vitamin D status improves clinical outcomes in FEP

by only a small degree, this treatment is cheap, simple to access (e.g. across the counter), relatively safe, and publicly acceptable.

We propose a randomised, double-blind, placebo-controlled, parallel-group trial of Vitamin D supplementation compared to placebo in people presenting with their first episode of psychosis.

4. Trial Objectives and Design

4.1. Trial Objectives

Primary Objectives:

To determine whether the addition of 120,000 IU monthly of vitamin D (cholecalciferol) supplement to standard treatments is more efficacious than placebo in improving outcomes (Positive and Negative Syndrome Scale Total score) at 6 month follow-up in those with First Episode Psychosis

Secondary Objectives:

To examine PANSS Total score and related sub-scores (Positive Scale, Negative Scale and General Psychopathology Scale) at 3 and 6 months, and a broader range of clinically-relevant outcomes; Global Assessment of Function (GAF), the Calgary Depression Scale, cardiovascular risk markers (Waist circumference, BMI, HBA1c, Total Cholesterol & CRP) and 25(OH)D concentrations at 6 months
We will examine primary and secondary outcomes in (a) all participants and (b) in a subgroup of the sample with suboptimal vitamin D concentrations at baseline (except for the secondary outcome of 25(OH)D concentrations which we will examine in all participants only).

Tertiary Objectives:

To collect data on inflammatory/immune markers

4.2 Trial Endpoints

Primary endpoint:

Total PANSS score at 6 month follow-up.

Secondary endpoints:

- Total PANSS score at 3 months
- PANSS Positive Scale subscore at 3 and 6 months
- PANSS Negative Scale subscore at 3 and 6 months
- PANSS General Psychopathology Scale subscore at 3 and 6 months
- Global Assessment of Function (GAF) at 6 months
- Calgary Depression Scale (CDS) at 6 months
- Waist circumference (cm) at 6 months
- BMI (kg/m2) at 6 months
- HbA1c (mmol/mol) at 6 months
- Total Cholesterol (mmol/L) at 6 months

- CRP (mg/L) at 6 months
- 25(OH)D Concentrations at 6 months

Tertiary endpoint:
Descriptive and comparative analysis of inflammatory/immune markers

4.3 Trial Design

We propose a randomised, double-blind, placebo-controlled, parallel-group trial of Vitamin D supplementation compared to placebo in people presenting with their first episode of psychosis. Study participants will be on active study treatment for a maximum of 6 months – for specifics on time points please refer to sections 4.3 and 7.1.

4.4 Trial Flowchart

Inclusion criteria

a) Aged between 18-65 years including women of child-bearing age
b) Diagnosis of functional psychosis defined according to ICD-10 criteria
c) Willing to refrain from taking multivitamins or non-study vitamin D supplements (including cod liver oil), that exceed 400IU/day of vitamin D throughout the study
d) Patients who are willing to give a vitamin D blood sample
e) Patients who are able to and have given written informed consent

Exclusion criteria

a) Known intolerance of Vitamin D2 or D3 or known allergy to any of the trial medications
b) Those who are currently taking vitamin D supplements at a dose exceeding 400IU/day.
c) Those who have taken cardiac glycosides; calcium channel blockers; or oral, intramuscular, or intravenous corticosteroids; bendroflumethiazide,; isoniazid, or rifampicin in the past one month
d) Known active tuberculosis, sarcoidosis, hypo or hyperparathyroidism, past or present nephrolithiasis (renal stones), suspected or diagnosed hepatic or renal dysfunction, any malignancy other than non-melanoma skin cancer not in remission for ≥ 3 years, calcium disorders
e) Baseline corrected serum calcium > 2.6mmol/L
f) Patients with known history of hypercalcaemia
g) Pregnant or breast-feeding women and women planning a pregnancy
h) Patients lacking the capacity to provide written informed consent
i) Those who do not have sufficient English to complete the core assessments with the available assistance

Screening, consent and baseline measures

↓

Randomisation 1:1
(where baseline bloods confirm eligibility)
(n=240)
Stratified by ethnicity

↓

Vitamin D | **Placebo**

↓

BEGIN INTERVENTION
Vitamin D: Active treatment with Cholecalciferol (Vit D_3) 120,000 IU/monthly, given orally as Vigantol® drops

Placebo: Identical bottles containing an organoleptically matched triglyceride oil, given monthly as oral drops

↓

Visit assessment dates:
- 3 months post randomisation (safety and PANSS)
- 6 months post randomisation (safety, clinical blood tests, and full assessment)

↓

Primary Outcome:
PANSS total score at 6 months
Secondary outcomes:
PANSS total score at 3 months
PANSS sub-scores (PANSS Positive scale, PANSS Negative scale and PANSS General Psychopathology scale) at 3 and 6 months
Global Assessment of Functioning (GAF) at 6 months
Calgary Depression Scale (CDS) at 6 months
Cardiovascular risk markers (Waist circumference, Body Mass Index, HBA1c, Total Cholesterol and CRP) at 6 months
25(OH)D concentrations

5. Trial Medication

5.1 Investigational Medicinal Product

The basis for the Investigational Medicinal Products is Vigantol® oil (by Merck GmbH). Vigantol® is an oral liquid where 1mL solution contains 0.5 mg Cholecalciferol, equivalent to 20,000 IU vitamin D3.

In the DFEND trial, eligible patients will be randomised to receive either vitamin D3 120,000IU or placebo once monthly for 6 months in a 1:1 ratio.

The comparator drug of Vigantol® oil will be achieved by using an organoleptically matched triglyceride oil (Miglyol® 812 oil) for the placebo. Both active and placebo will be primarily packaged in identical glass bottles (fill volume 8mL). All medication will be labelled according to Annex 13 guidelines.

Active and placebo treatment will be administered orally as 6mL given in a graduated oral syringe by a trained researcher.

Guy's & St Thomas' Pharmacy Manufacturing unit is responsible for arranging the IMPs' manufacture, randomised labelling, packaging and final QP release for clinical trial use. Please refer to Summary of Product Characteristics for Vigantol® Oil and the Investigational Medicinal Product Dossier (IMPD) for more details about the active and placebo IMPs.

5.2 Dosing Regimen

The active treatment - Cholecalciferol (vitamin D3), 120,000 IU per month (equivalent to 4,000 IU per day), will be given orally as Vigantol® oil (by Merck GmbH) cholecalciferol dispersed in triglyceride oil as vehicle; containing 20,000 IU cholecalciferol per 1ml drop, and administered as 6mL given in a graduated oral syringe by a fully trained member of the research team. The placebo will be administered by the same trained research team and with the same method. Both will be administered monthly for 6 months.

5.3 IMP Risks

The Summary of Product Characteristics (SmPC) will be the Reference Document.

Contra-indications - Vigantol Oil must not be used in:

- Hypersensitivity to the active substance cholecalciferol or to any of the excipients
- Hypercalcaemia and/or hypercalciuria
- Hypervitaminosis D
- Renal osteodystrophy with hyperphosphatemia

Special warnings and special precautions for use

- During long-term treatment with a daily dose exceeding 1000 IU vitamin D the serum calcium values must be monitored.

- Special attention should be paid to patients treated with thiazide diuretics, to patients with nephrolithiasis history, to those suffering from sarcoidosis and during pregnancy. Special attention is also requested in patients with atherosclerosis, cardiac disorders, renal disorders and hyperphosphatemia. It is recommended to monitor levels of calcium, phosphates, magnesium, cholesterol (increases with higher doses) and alkaline phosphatase in serum

Particular caution is recommended:

- When taking additional doses of vitamin D, e.g. via other medicinal products
- In pseudohypoparathyroidism particular attention must be paid to signs of intoxication. There may be phases of normal sensitivity to vitamin D so that the dose has to be adjusted accordingly
- In pseudohypoparathyroidism after thyroid surgery Vigantol must be stopped as soon as recovery of the parathyroid glands is observed, in order to avoid vitamin D intoxication

Interaction with other medicinal products and other forms of interaction

- Digitalis (cardiac glycosides): Oral administration of vitamin D3 may increase the efficacy and toxicity of digitalis. Regular medical supervision is required including assessment of ECG, serum calcium, and possibly also digoxin plasma levels.
- Thiazide diurectics (e.g. benzothiadizine): The urinary calcium excretion may be reduced and the risk of hypercalcaemia may be increased.
- Barbiturates (e.g. phenobarbital) or phenytoin: plasma concentrations of 25-OH D may be decreased and metabolism to inactive metabolites may be increased,
- Corticosteroids: may decrease the efficiency of vitaminD3
- Vitamin D3 metabolites or analogues: It is recommended to combine with Vigantol only in exceptional cases and with monitoring of serum calcium levels.
- Rifampicin and isoniazid: may increase the metabolism of vitamin D3 and reduce its effectiveness.
- Large doses of vitamin D may cause hypercalcemia, increase cholesterolemia, lower alkaline phosphatase activity, tend to cause alkalosis
- Administration of medicaments containing magnesium aluminium (some antacids) is not recommended.

Fertility, pregnancy and lactation

During pregnancy and lactation sufficient intake of vitamin D is required. No data on acute or chronic toxicity of vitamin D levels on pregnant women are available. Hypercalcaemia caused by vitamin D overdosing during pregnancy may be associated with increased susceptibility to the effects of vitamin D, suppression of parathyroideal function, mental retardation, and aortic stenosis in child. On the other hand, significant hypovitaminosis D and osteomalacia in women may cause fetal or neonatal rachitis, hypoplasia of enamel and hypocalcemia. No teratogenic effects have been proven on laboratory animals after the doses 6-12 times higher than human doses have been administered. However, there are many cases when patients with hyperparathyroidism used 100,000 IU of cholecalciferol a day and delivered a healthy child have

also been recorded. Therefore, administration of larger doses of vitamin D3 on pregnant women should be considered by a specialist.

Pregnant women or those planning pregnancy will not be eligible to participate in the trial. We will not make it mandatory that women of child-bearing age are on contraception however we will monitor pregnancy status throughout the trial by reviewing pregnancy status with all female participants and administering a routine urine dipstick pregnancy test. We acknowledge that overdose of vitamin D must be avoided during pregnancy, because prolonged hypercalcaemia can lead to physical and mental retardation, supravalvular aortic stenosis and retinopathy of the child. To address this safety issue, we have specifically chosen an amount of Vitamin D considered to be safe for use in pregnant women (Vigantol® 120,000 IU/month equivalent to 4000 IU/Day). We will continue to screen for pregnancy before giving each monthly dose. If we detect pregnancy in any female participants during the course of the trial, they will be withdrawn from the treatment. However, pregnancy tests will not be done for female participants who are permanently sterile or who are post-menopausal (no menses for 12 months without an alternative medical cause).

We will also ensure that all patients are made aware of the symptoms of hypercalcaemia (loss of appetite, nausea and vomiting, constipation and abdominal pain, increased thirst and frequent urination, fatigue, weakness, and muscle pain). If any of these symptoms are detected in between dosing visits, patients will be advised to contact their GP, so that their GP can arrange for a blood test for hypercalcaemia (and report hypercalcaemia as a suspected adverse reaction to the Trial Coordinating centre). First line treatment for hypercalcaemia is simple rehydration but if this is not effective then pharmacological interventions can be used. The GP will advise of this. The research team who administer the monthly IMP/placebo dose will also ask the patient if they have experienced any of these symptoms since they were last seen by the research team, this will be done routinely every month before any IMP/placebo administration. Should these symptoms be reported at a scheduled visit, a blood test will be offered to the patient to check calcium levels and ensure patient-safety.

Participants will be asked to refrain from taking vitamin D supplements (including multivitamins containing vitamin D); however, supplementation of up to 400IU/day will be permitted throughout the trial, as recommended by Public Health England.

5.4 Drug Accountability

Vigantol® Oil is a vitamin based supplement and its main component cholecalciferol is a natural compound that is synthesized by the skin after exposure to UVB sunlight. We are raising the levels of a compound to which participants are naturally exposed in their everyday lives. This trial is classified as a Type B Trial for regulatory purposes.

Full IMP accountability will be maintained throughout the trial. IMP administration will be logged and then each bottle will be safely disposed of immediately after use. Used bottles will

not be returned to Pharmacy but will be disposed of in a sharps bin. Unused bottles will be returned to pharmacy and safely destroyed.

5.5 Storage of IMP

IMP/Placebo will be stored in Pharmacy according to manufacturer's guidelines.

5.6 Subject Compliance

Participants will be given IMP/Placebo orally by one of the research team with appropriate training and delegation to do so. The researcher will complete a source data worksheet to state the time, date, amount of IMP administered and the administering researcher for each dose administered throughout the study period.

As previously mentioned, the participants will be given the IMP/placebo once a month for 6 months. A dosing visit will be scheduled each month for a total of 6 months, where the initial dosing date, acts as the anchor date for all future visits (i.e. the first appointment should be scheduled a month after from the initial dose, and so on). A minimum of a 24-day interval between doses must be adhered to throughout the study to ensure that patients are not dosed too closely. A visit window of -2/+2 weeks from the anchor date will be set to allow room for missed/rescheduled appointments. If a dose is missed, the participant will be allowed to carry on with the trial and the next dose of treatment (either IMP/placebo) will be administered at the next scheduled time point; in such instances a protocol deviation will be logged.

We will seek permission from participants to voluntarily provide contact details of a close relative or friend plus at least 2 means of contact including email address, mobile phone number, landline phone number, in order to improve the likelihood of contacting patients at future follow-up points. We will note which is their preferred method. This information will be stored on a separate contact details form and will be kept in a locked file by the team administrator. We will make it explicitly clear that the contact details of a significant other will only be used in situations whereby we are unable to contact the participant in the first instance.

In the event that a participant decides they no longer wish to take the treatment (IMP/placebo) they will be encouraged to continue with the study, if agreeable they will be followed up at the main assessment points (6 months) but routine safety checks will not be offered, except for any due after their last dose of IMP and no drug will be administered. Participants are free to drop out from the study at any time.

For the follow-up assessment at 6 months a target visit window of -4/+6 weeks will be set to ensure follow-up data is collected. If the data can only be collected outside of this target visit window, it should done anyway. For routine safety blood visits a -2 week window will be adhered to but an upper time frame will not be specified for collecting the safety bloods as these must be performed. Data collection will end at end of study defined in section 6.6.

5.7 Concomitant Medication

No restrictions are placed on the prior or concomitant interventions (medications or therapies, rescue or subsequent treatments) allowed, other than we ask the participants to refrain from taking vitamin D supplements (including multivitamins that contain vitamin D) that exceed 400IU/day for the duration of the trial. At each treatment visit (once monthly for 6 months) and at the final assessment, researchers will ask the participant if they are taking any additional vitamin D supplements. If the participant indicates that they have been taking additional vitamin D supplements that exceeds 400 IU/day, a blood sample will be collected and tested for calcium. If the corrected calcium is above normal range, a parathyroid test will be performed. The participant will be asked to refrain from taking further vitamin D supplements that exceed 400IU/day and if they decline to do so, they will be encouraged to stay involved in the study for the full duration but no more IMP/placebo will be administered.

6. Selection and Withdrawal of Subjects

6.1 Inclusion Criteria

All patients within EIS and FEP inpatient units who meet the following criteria will be invited to participate in the study:

a) Aged between 18-65 years old including women of child-bearing age
b) Having a diagnosis of functional psychosis FEP defined according to ICD-10 criteria for psychosis (codes F20-29 and F30-33)
c) Willing to agree to refrain from taking multivitamin or non-study vitamin D supplements that exceed 400IU/day throughout the study
d) Willing to give a blood vitamin D sample at baseline
e) Patients who are able and have given written informed consent

6.2 Exclusion Criteria

a) Known intolerance of Vitamin D2 or D3 or known allergy to any of the trial medications
b) Those who are currently taking vitamin D supplements at a dose exceeding 400IU/day
c) Those who have taken cardiac glycosides; calcium channel blockers; or oral, intramuscular, or intravenous corticosteroids; bendroflumethiazide; isoniazid; or rifampicin in the past one-month
d) Known active tuberculosis, sarcoidosis, hypo or hyperparathyroidism, past or present nephrolithiasis (renal stones), suspected or diagnosed hepatic or renal dysfunction, any malignancy other than non-melanoma skin cancer not in remission for ≥ 3 years, calcium disorders
e) Baseline corrected serum calcium > 2.6mmol/L
f) Patients with known history of hypercalcaemia
g) Pregnant or breast-feeding women and women planning a pregnancy
h) Patients lacking the capacity to provide written informed consent
i) Those who do not have sufficient English to complete the core assessments with the available assistance

6.3 Selection of Participants and informed consent

The study population is patients in their first episode of psychosis (FEP) defined as first presentation to services within the last three years. Participants will be recruited from NHS Trust research sites (including Early Intervention Services, Home Treatment Teams, Inpatient Units, Community Teams and Participant Identification Centres (PIC)). Where participants are recruited from PICs consent and other trial activities will be undertaken at the host research site. To be eligible for entry into the trial individuals must satisfy all of the inclusion criteria and none of the exclusion criteria. The eligibility criteria are purposefully minimal to facilitate the enrolment process and to maximise generalisability of the trial results, namely individuals diagnosed with first episode psychosis attending clinical services.

An ICH-GCP trained clinician (research nurse, doctor or allied health professional) will be responsible for obtaining written informed consent from all trial participants prior to baseline assessment. Eligibility will be confirmed before randomisation by the PI or delegated study doctor **only**.

Subjects will be offered £80, in total, as reimbursement for the time they give to participating in the trial. £20 will be offered at baseline, £20 at the 3 month safety check, and a final £40 after the final assessment. Reasonable travel expenses incurred whilst traveling both to and from study appointments will also be reimbursed. Participants active in the study at the time of this change will receive a total of £80. The new payment schedule will be adhered to for baseline and Month 3, and any extra amount will be paid at the Month 6 assessment.

6.4 Randomisation Procedure / Code Break

6.4.1 Randomisation

An online randomisation service will be provided by King's Clinical Trials Unit. Randomisation will take place after collection of baseline measures. The unit of randomisation will be the individual participant. Randomisation will be at a 1:1 ratio and the method of randomisation will be stratified randomisation with randomly varying block sizes of 2-4 where the stratification factor is ethnicity (with 2 levels: White, Other).

We chose to balance treatment arms by ethnicity as this is thought to predict outcomes. There are no known predictors of PANSS in our target population. However, blood vitamin D concentration varies by ethnicity; and we hypothesize that blood vitamin D concentration mediates any effect of vitamin supplement. Thus ethnicity was chosen as a randomisation stratifier.

Each active and placebo IMP bottle will have a unique identification number. Each patient will be assigned a unique Patient Identification Number (PIN) which will be used throughout the duration of their treatment. To request randomisation service user accounts and/or email notifications of randomized patients contact the DFEND trial manger on dfend@kcl.ac.uk.

The procedure is as follows:

1. Informed Consent is obtained
2. Baseline blood samples are taken as part of baseline assessment to confirm eligibility
3. Once the participant is found to be eligible, a trained/delegated researcher will input registration/eligibility information into the EDC Macro v4.0 to obtain a PIN.
4. This PIN and other key identifying information (Name/DOB/ethnicity) is then inputted by the trained researcher into the randomisation online system.
5. Once the information is submitted, the online system, which is managed by the King's Clinical Trials Unit (KCTU), then will randomly allocate the patient to a study arm and notify the unblinded members of staff, which are currently: the CRA from the KHP CTO (sponsor office), the trial pharmacy staff and the emergency code breaking service. The blinded members of staff should include all research teams at site level including the study site PI.

If any errors are made in the request (e.g. incorrect patient initials, date of birth or stratification factors), as soon as the error is noticed by the researcher inputting the information, they should contact the trial manager and the randomisation service team on randomization.request@kcl.ac.uk and it will be logged on the system. A randomisation cannot be 'undone' where incorrect stratification factors have been used, so care will be taken when requesting randomisation. However information about errors can be recorded alongside the randomisation data for that patient, so that this key information is not missed.

6.4.2 Emergency Code Break

24hr Emergency Code Break and Medical Information will be provided by ESMS Global Ltd. Each randomised subject will be provided with a patient card detailing code break telephone numbers and emergency contact details. Subjects will be requested to carry this card with them at all times whilst participating in the trial. The only people authorised to make emergency enquiries to the code breaking service are healthcare professionals who have direct responsibility for the care of the trial subject concerned (clinical trial investigators, pharmacists and the KHP CTO staff are also authorised to request unblinding). The subjects themselves, their relatives or other members of the public are not authorised users and if they use the number and request unblinding they will be directed to contact their trial team and/or local PI.

6.5 Withdrawal of Subjects

Participants have the right to withdraw from the study at any time for any (or no) reason. The investigator also has the right to discontinue patients from the study drug in the event of AEs, SAE's, SUSAR's, protocol violations, administrative or other reasons. It is understood by all concerned that an excessive rate of withdrawals can render the study un-interpretable; therefore, unnecessary withdrawal of patients should be avoided. Should a patient decide to withdraw from the study, all efforts will be made to report the reason for withdrawal as thoroughly as possible. Should a patient wish to discontinue the study drug only, efforts will be made to continue to collect follow-up data, with the permission of the patient.

In light of substantial changes to the study duration, all participants will be informed of changes made to the study initially by letter and again in person at the next patient visit. They will be

provided with a new patient information sheet, and will be invited to sign and date a new consent form. Those participants who do not agree to the new protocol changes will be withdrawn from the study.

Specifically, for those participants who originally consented to a 12 month trial and who have received more than 6 monthly doses of the IMP, we will still perform the 9 month safety blood test and conduct the 12 month follow up assessments as originally planned (see Table 1). The only change will be that these participants will receive no further doses of IMP.

For those participants who originally signed up to a 12 month trial and who have received less than 6 monthly doses of the IMP until 02.06.17, we will invite patients to give consent to the new consent form (V8a 02.06.17) which states that the trial will end at the 6 month follow-up assessment mark, after which no further IMP will be dosed. We will honour the original total planned payment described in ICF v7 02.12.2016 and PIS v8 02.12.2016: £5 at the 9 month safety check and £10 at the 12 month follow-up assessment. This total of £15 will be paid at the 6 month visit.

6.6 Expected Duration of Trial

The end of the trial will be defined as the last patient's post 6 month visit telephone call. Each participant will remain on the trial until they have each completed the post 6-month visit telephone call. The study will run from December 2015 until December 2019 to ensure that each participant has 6 months of exposure to either the active or placebo treatment.

7. Trial Procedures

7.1 By Visit

Participants will receive a monthly dose of either Vigantol® Oil 120,000 IU per month (equivalent to 4,000 IU per day) or placebo administered by trained members of the research team.

Table 1: DFEND Trial Outcome Measures at Different Time-points

**STUDY PERIOD**	**SCREENING AND BASELINE**	**RANDOMISATION**	**TREATMENT Vigantol®/Placebo**	**SAFETY CHECK**	**ASSESSMENT VISIT**	**FOLLOW UP PHONE CALL**	**SAFETY CHECK*******	**ASSESSMENT VISIT*******
**VISIT Name**	**Baseline VISIT**		**DOSING VISITS 1 – 6**	**3 MONTH VISIT**	**6 MONTH VISIT**	**POST 6 month CONTACT**	**9 MONTH VISIT**	**12 MONTH VISIT**
**TIMEFRAME**	**WEEK -2**	**WEEK 0**	**Monthly for 6months (Visit 1 is the anchor date, Visits 2 to 6: -2/+2 weeks from anchor date)**	**MONTH 3**	**MONTH 6 (Visit 6 assessment: -4/+6 weeks from anchor date)**	**POST 6 mth**	**9 MONTHS**	**12 MONTHS**
Eligibility & Informed consent	X							
Sociodemographics	X							
NOS (Duration of Untreated Psychosis)	X							
Current medication	X		X	X	X			
Medical history	X							X
Vitamin supplementation	X		X	X	X			
Anthropometrics	X				X			X
Blood sampling: vitamin D concentration (storage)	X				X			X
Blood sampling: incl. clinical, genetic, cardiovascular and inflammatory markers	X				X			X
Urine pregnancy test	X*		X*	X*	X*			
Smoking habits	X				X			X
PANSS	X			X	X			X
GAF	X				X			X
CDS	X				X			X
OPCRIT	X							
Sun Exposure questionnaire	X				X			X
IPAQ	X				X			X
SIMPAQ	X				X			X
Fitzpatrick Skin Type Questionnaire	X							
Vitamin D Food Frequency Questionnaire	X				X			X
Randomisation		X						
Adverse events recorded **	X		X	X	X	X	X	X
Blood sampling for calcium levels (including parathyroid hormone test if calcium is above normal range)	X		X***	X	X		X	
Administration of IMP or Placebo			X****	X****	X****			
Patient Medication Guess			X	X	X	X	X	X
Service Contacts Form	X				X			X

* In the event that it is not possible to obtain a urine sample, a blood HCG sample will be obtained and tested for pregnancy. No IMP will be administered without first ascertaining pregnancy status. Pregnancy tests will not be done for female participants who are permanently sterile or who are post-menopausal (no menses for 12 months without an alternative medical cause).
** AEs collected from consent. Patients will be phoned 28 days post the last dose of treatment at 6 months to check for any adverse events. At dosing visits 1 – 6, we will assess adverse events in person with the participant at their scheduled visit. If no IMP is given at the Month 6 visit, no follow-up call will be needed. Should a participant withdraw from the study, AEs will need to be collected up to 28 days after their last dose.
*** Blood sampling for calcium levels (including parathyroid hormone test) will only be performed at these time points if patient reports nausea and vomiting.
**** The IMP is administered once a month, every month for 6 months.
***** The 9-month safety check and 12-month assessments will only be performed on those patients who have received more than 6 monthly doses of the IMP prior to the protocol change dated (02.06.17). However, the IMP/placebo will no longer be administered after 6 months.

At the assessment visits (Baseline and 6 months) participants will be required to complete the following assessments:

a) Mental health care: A list of current medications; medication history, including antipsychotic, antidepressant, mood stabilisers, non-psychiatric medications and non-prescription drugs and vitamin supplements will be gathered at each time point. We will establish the duration of untreated psychosis (DUP) at baseline using the Nottingham Onset Schedule (NOS). Use of key NHS services contact questionnaire will measure the total number of hospital admissions and the amount of contact with the home treatment team throughout the duration of the trial. This will be recorded from the time of consent up to the Month 6 assessment. Data will be collected from patients at their final assessment and checked against medical notes. For patients who are lost to follow-up, this data will be obtained from medical notes only.

b) Biomedical / Cardiovascular status (secondary endpoint) will be measured at each time point as: waist circumference, weight, height (for BMI), blood pressure, heart rate (pulse) (Anthropometric measures), along with blood measures of cardiovascular risk / inflammatory markers and smoking habits. (Measures which form part of routine clinical care will be shared with the clinical team if the patient wishes).

c) Mental health status (primary and secondary endpoints): the Positive and Negative Syndrome Scale (PANSS); Global Assessment of Functioning (GAF). The GAF will be rated first, from clinical notes at the time of first presentation to services for psychosis and then again at baseline to ensure an accurate representation of functioning at both the onset of illness and at the time of consent; Calgary Depression Scale (CDS); OPCRIT (Operational Criteria checklist) will be performed during the trial to ensure that we have a researcher-led clinical rating independent of the diagnosis retrieved from clinical notes at the time of screening.

d) Urine pregnancy tests in females, monthly, at each treatment visit (If positive, IMP will be discontinued). In the event that it is not possible to obtain a urine sample, a blood HCG sample will be obtained and tested for pregnancy. No IMP will be administered without ascertaining pregnancy status. Pregnancy tests will not be done for female participants who are permanently sterile (e.g. who have undergone hysterectomy, bilateral salpingectomy or bilateral oophorectomy) or who are post-menopausal (no menses for 12 months without an alternative medical cause).

e) Levels of sun exposure, including type of clothing worn, levels of physical activity (IPAQ) and SIMple Physical Activity Questionnaire (SIMPAQ), skin type (Fitzpatrick Skin Type questionnaire), vitamin D food frequency questionnaire (Other influences on Vitamin D levels).

f) Adverse event assessment will be systematically addressed during the monthly contact with the participant. Additionally, at the 3 month visit a blood sample will be taken to monitor corrected serum calcium (Ca^{2+} levels or "bone screen"). A parathyroid hormone

test (PTH) will be performed on the same blood sample if Ca^{2+} is above normal range. If participants report nausea and vomiting between visits, they will be advised to contact their doctor for a Ca^{2+} blood test.

g) At each dosing visit and during the follow-up phone call, participants will be asked whether they think they were assigned to receive the treatment (vitamin D) or the placebo, and their reason for this. This is to record how well the blind was maintained throughout the study

7.2 Laboratory Tests

All blood samples will be obtained by a trained phlebotomist.

Blood will be taken for 25OHD concentration at baseline and 6 months but this assay will not be performed until the trial is complete. This is to ensure that researchers and clinicians are blind to baseline vitamin D status and that the patients will be offered standard clinical treatment. Additionally there is clinical equipoise regarding vitamin D supplementation in this group (i.e. the uncertainty of the intervention's efficacy versus the potential that leaving participant's vitamin D deficient will cause harm). This strategy has been employed successfully by one of the investigators (Fiona Gaughran) elsewhere (Gaughran et al., 2007). At the end of the study, once all data is analysed we will inform the participant's respective clinical team and/or GP of their vitamin D levels at last testing, noting that it may be some time after that test. Appropriate recommendations for post-study vitamin supplementation will be made by patient's GP or clinical team. Once the study is complete, serum samples will be shipped to the funder (Stanley Medical Research Institute) as part of the funding agreement, in an anonymous format. The Biomedical Research Centre (BRC) will store this sample until it is ready to be shipped to America. Clinical markers for cardiometabolic and general health will be tested from the same bleed (and shared with the clinical team with the patient's permission), and samples stored to later test for immune/inflammatory markers. Safety tests include calcium and if indicated parathyroid hormone test.

7.2.1 DNA

From the blood samples collected at baseline, in patients who have additionally consented to genetic testing, we will also extract DNA , to explore possible interactions between common variants in vitamin D pathways and clinical outcomes (as been recently demonstrated in a study of vitamin D supplementation for Parkinson's Disease - Suzuki et al., 2013). Patients will also have the option to have their DNA stored in the NIHR BioResource Centre Maudsley and the NIHR BioResource. The biobanking and long-term storage of DNA will be described in the BioResource's PIS and subject to separate consent.

7.2.2 RNA

Patients consenting to the study at an early stage will be asked to give a blood sample for RNA. RNA analysis at baseline and 6 months will be used to explore gene expression after supplementation (Hossein-nezhad, 2013). These blood samples will be collected at the same time as the baseline/Month 6 vitamin D samples, in separate blood tubes. The samples will be

delivered directly to the BRC Biobank at the SGDP (Social, Genetic and Developmental Psychiatry) building at King's College London.
Patients enrolling at a later stage and consenting to ICF v10 (dated 09.01.2019) will no longer be asked to provide RNA. Enough paired samples have been collected and the added burden to patients should be prevented.

8. Assessment of Efficacy

8.1 Efficacy Parameters

8.1.1 Primary Efficacy Parameters

Difference between trial arms in Positive and Negative Syndrome Scale (PANSS) total score at 6 months in all FEP patients.

8.1.2 Secondary Efficacy Parameters

The following measures will be assessed in all participants irrespective of vitamin D concentration:

- Positive and Negative Syndrome Scale (PANSS) at 3 months
- Positive and Negative Syndrome subscores (Positive Scale, Negative Scale and General Psychopathology Scale) at 3 and 6 months
- Global Assessment of Functioning (GAF) at 6 months
- Calgary Depression Scale at 6 months
- Cardiovascular risk markers (Waist circumference, BMI, HBA1c, Total Cholesterol & CRP) at 6 months

We will also measure the efficacy of vitamin D supplementation at 6 months as indicated by 25OHD blood concentration levels.

In addition to the whole sample, we will examine PANSS total scores at 3 and 6 months in a subsample of those with suboptimal baseline vitamin D levels.

8.1.3 Exploratory moderator analysis

Inflammatory markers will be measured and examined as a tertiary/exploratory efficacy parameter. This will not form part of the primary or secondary analyses. These post-hoc analyses will only become clear once the main objectives of the trial have been evaluated. The trial population for which we wish to investigate these markers as potential predictor markers (predictors of treatment effects) can only be defined once the investigators have a clear summary of where vitamin D is effective, i.e. in the entire (non-tested) population or the vitamin D insufficient / sufficient population. Interaction with treatment effects can be used to evaluate these variables.
Serum aliquots will be sent to the funder, Stanley Medical Research Institute in the USA, for future inflammatory marker analysis.

8.2 Procedures for Assessing Efficacy Parameters

Concerning efficacy, psychopathology will be assessed with the PANSS total score and related sub-scores, Global Assessment of Function (GAF) and Calgary Depression Scale, and 25OHD blood concentration levels at 6 month assessment visits.

Blood will be taken for 25OHD concentration at baseline and 6 months but this assay will not be performed until the trial is complete.

Concerning follow-up, all participants will be seen by trained research staff each month for the administration of the intervention. Research staff will assess outcomes at baseline, 3 (PANSS only) and 6 months, including anthropometric/cardiovascular assessments. With prior consent from the participant clinically relevant measures will be shared with the clinical teams to minimise duplication of assessments.

Primary outcomes will be assessed at 6 months in order to reduce the influence of attrition. Based on past research undertaken in London, we expect that up to 20% of FEP will drop out by 6 months.

Concerning safety, at study entry, all patients will be assessed with a physical examination, including weight, blood pressure, and pulse rate. Bloods will be taken at baseline and 3 months, in order to assess serum calcium (corrected for serum albumin) and parathyroid hormone (PTH) where indicated and at additional times if there is evidence of symptoms of hypercalcaemia. Side effects will be systematically assessed at each month prior to administration of the intervention (IMP/placebo).

9. Assessment of Safety

9.1 Specification, Timing and Recording of Safety Parameters

Any adverse event that occurs between the time of consent to the study through to 28 days following their last dose will be recorded via a telephone conversation with the participant. Should a participant not receive the IMP dose during final assessment, for example if outside of the dosing window, a follow-up telephone call will not be needed. Should a participant withdraw from the trial, AEs will be recorded up to 28-days after their last dose. Every reasonable effort ~~Three attempts, approximately one week apart,~~ should be made to contact the participant for their 28-day post-last dose telephone call to record any AEs. The investigators will assess whether the adverse event may be related to the subject’s participation in the study.

Particular attention will be paid to symptoms of hypercalcemia which are: loss of appetite, nausea, vomiting, constipation, abdominal pain, increased thirst, frequent urination, fatigue, weakness, and muscle pain.

In addition, the following assessments will be used to determine subject safety during the study at baseline and 3 months:

1. Corrected serum calcium (Ca^{2+} levels or "bone screen"), will be performed at baseline, and three months and more frequently if nausea and/or vomiting occur.
2. A parathyroid hormone test (PTH) will be performed on the same blood sample if Ca^{2+} is above normal range. If participants report nausea and vomiting between visits, they will be advised to contact their doctor for a Ca^{2+} blood test.
3. A urine dipstick pregnancy test (beta-HCG) will be performed for all females at baseline. Female participants of child-bearing age refusing a pregnancy test at baseline will be excluded from participating in the trial. At each treatment visit (monthly for 6 months) a urine dipstick pregnancy test will be performed to determine pregnancy status. Pregnancy tests will not be done for female participants who are permanently sterile or who are post-menopausal (no menses for 12 months without an alternative medical cause). If the urine test is positive, the participant will be withdrawn from the trial medication but encouraged to remain in the study. At 3 and 6 month visits the researcher will perform a urine dipstick test to ascertain pregnancy status. In the event that it is not possible to obtain a urine sample, a blood HCG sample will be obtained and tested. No dose of IMP will be administered to female participants without first checking pregnancy status (except for patients who are permanently sterile or post-menopausal, as described above).

9.2 Procedures for Recording and Reporting Adverse Events

The Medicines for Human Use (Clinical Trials) Regulations 2004 and Amended Regulations 2006 gives the following definitions:

Adverse Event (AE): Any untoward medical occurrence in a subject to whom a medicinal product has been administered including occurrences which are not necessarily caused by or related to that product.

Adverse Reaction (AR): Any untoward and unintended response in a subject to an investigational medicinal product which is related to any dose administered to that subject.

Unexpected Adverse Reaction (UAR): An adverse reaction the nature and severity of which is not consistent with the information about the medicinal product in question set out in the summary of product characteristics (SmPC) for that product (for products with a marketing authorisation)

Serious adverse Event (SAE), Serious Adverse Reaction (SAR) or Unexpected Serious Adverse Reaction (USAR): Any adverse event, adverse reaction or unexpected adverse reaction, respectively, that:

- Results in death;
- Is life-threatening;
- Required hospitalisation or prolongation of existing hospitalisation
- Results in persistent or significant disability or incapacity;

- Consists of a congenital anomaly or birth defect.

Suspected Unexpected Serious Adverse Reaction (SUSAR) – A Suspected Unexpected Serious Adverse Reaction is a serious adverse drug reaction, the nature and severity of which is not consistent with the information about the medicinal product in question set out:

- In the case of a product with a marketing authorisation, in the summary of product characteristics for that product.
- In the case of any other investigational medicinal product, in the investigator's brochure relating to the trial in question.

Important Medical Events (IME) & Pregnancy
Events that may not be immediately life-threatening or result in death or hospitalisation but may jeopardise the patient or may require intervention to prevent one of the other outcomes listed in the definition above should also be considered serious.
Although not a serious adverse event, any unplanned pregnancy will also be reported via the SAE reporting system.

Reporting Responsibilities
King's College London and South London and Maudsley NHS Foundation Trust have delegated the delivery of the Sponsor's responsibility for Pharmacovigilance [as defined in Regulation 5 of the Medicines for Human Use (Clinical Trials) Regulations 2004] to the King's Health Partners Clinical Trials Office (KHP-CTO).

All SAEs, SARs and SUSARs will be reported immediately by the Chief Investigator (and certainly no later than 24hrs) to the KHP-CTO in accordance with the current Pharmacovigilance Policy.
The KHP-CTO will report SUSARs to the regulatory authorities (MHRA, competent authorities of other EEA (European Economic Area) states) in which the trial is taking place.

The Chief Investigator will report to the relevant ethics committee. Reporting timelines are as follows:

SUSARs which are fatal or life-threatening must be reported not later than 7 days after the sponsor is first aware of the reaction. Any additional relevant information must be reported within a further 8 days.

SUSARs that are not fatal or life-threatening must be reported within 15 days of the sponsor first becoming aware of the reaction.

The Chief Investigator and KHP-CTO (on behalf of the co-sponsors), will submit a Development Safety Update Report (DSUR) relating to this trial IMP, to the MHRA and REC annually.

9.2.1 Adverse events that do not require reporting

All SAEs will be reported for this trial except hospitalisations due to deterioration in mental state, as we anticipate a high proportion of our participants will be hospitalised for this reason during the trial.

The period for AE reporting is from consent to 28 days post last IMP administration. All AEs will be reported.

9.3 Treatment Stopping Rules

The trial may be prematurely discontinued by the Sponsor, Chief Investigator or Regulatory Authority on the basis of new safety information or for other reasons given by the Data Monitoring Committee, Trial Steering Committee, regulatory authority or ethics committee concerned.

If the trial is prematurely discontinued, active participants will be informed and no further participant data will be collected. The Competent Authority and Research Ethics Committee will be informed within 15 days of the early termination of the trial.

10. Statistics

10.1 Sample Size

A total of 240 first episode psychosis patients will be recruited over a 2 year period.

Power Calculation

Based on recently collected PANSS Total score in FEP patients collected as part of the Physical Health and Substance Use Measures in first episode Psychosis (PUMP) study (n = 190), the mean (and standard deviation) score was 58.1 (15.0) units. Based on the number of new cases presenting to FEP services, and based on the annual counts of FEP patients recruited into similar studies, we predict that we will recruit and randomise 120 patients per year (i.e. 240 over two years).

The primary intention to treat (ITT) analyses will compare two equally sized treatment arms, treatment or placebo on the PANSS scale at 6 months post-randomisation. For the power analyses, we modelled two plausible scenarios. For the primary outcome (6 month follow-up), we assumed a 20% attrition proportion. After 20% attrition the effective sample size is 192 (96 in each trial arm).

Based on alpha = 0.05 and power = 80%, samples between 200 and 180 participants will be able to confidently detect mean PANSS total score group differences of between 6 and 6.3 units. This equates to a standardised effect of size of approximately 0.4 to 0.42.

Considering 90% power with the same power assumptions, we will be able to detect mean PANSS total score group differences of between 6.9 and 7.3 units (standard effect size from 0.46 to 0.49). Thus, we should be able to detect moderate effect sizes if present.

As a secondary objective, we also wish to estimate the average treatment effect in those participants who meet the vitamin D deficiency threshold. We assume 60% of the FEP

population have vitamin D concentrations below 50 nmol/L. At 6 months after 20% attrition (sample now = 192) and 60% who meet the vitamin D threshold, this will result in a projected sample size of 115.

Based on alpha = 0.05 and power = 80%, samples between 120 and 100 participants will be able to confidently detect mean PANSS total score group differences of between 7.7 and 8.5 units This equates to a standard effect of size of 0.52-0.57. Considering 90% power with the same power assumptions, we will be able to detect mean PANSS total score group differences of between 9.8 and 8.95 units (standardised effect size from 0.60 to 0.65).

10.2 Analysis

The primary analyses of efficacy will be based on the intention-to-treat sample, utilising all available follow-up data from all randomised participants. The significance level will be 5% (2-sided) for all specified main and secondary analyses with estimates and confidence intervals presented for all effects.

All descriptive analyses, recruitment rate, consent rate, loss to follow-up, departures from randomised treatment and the prevalence of serious adverse events post-randomisation will be reported and summarised by treatment arm over the course of the study. All causes of withdrawal from randomised treatment will be reported. BMI will be calculated using the following formula: weight (kg)/height $(m)^2$.

The main objective of the formal statistical analyses is to assess the effect of vitamin D supplementation on the primary outcome PANSS total Score at the 6 month follow-up time point. To this end linear mixed modelling (LMM) will be employed.

In such models the outcome variable measured at the post treatment time points (here 3 and 6 months) features as the dependent variable with, treatment arm (vitamin D or placebo), time (3 or 6 months), baseline PANSS and randomisation stratifier (ethnicity) included as covariates. The model will also contain a subject-varying random intercept to account for any correlation between the repeated measures.

Treatment effects on secondary outcomes will be assessed using similar modelling techniques, employing generalisations to non-normal data where necessary.

We will also estimate subpopulation (baseline vitamin D insufficient and sufficient) specific treatment effects for the primary outcome and secondary outcomes except for 25OHD blood concentration levels by including relevant interaction terms between baseline vitamin D insufficiency status and treatment group in the statistical models.

We expect there to be some missing data in the post-treatment outcomes variables. The LMM analyses are based on maximum likelihood and will provide valid inferences under a missing at random (MAR) missingness mechanism. We will explore predictors of missingness, if deemed suitable for adjustment we will include these as explanatory variables in the analyses. If post

randomisation variables are identified a Multiple Imputation [White, I.R., P. Royston, and A.M. Wood, *Multiple imputation using chained equations: Issues and guidance for practice.* Statistics in Medicine, 2011. **30**(4): p. 377-399] model will be considered instead.

For those participants who initially signed up to a 12-month trial, we will not perform any statistical analysis on the 12-month data, but we believe it is ethically correct to assess those patients at the 12 month follow up visit as originally planned.

11. Trial Steering Committee

The terms of reference of the DFEND Trial Steering Committee are as follows:

1. To approve the final protocol for the DFEND trial. If changes are required to the final protocol, to agree these changes before they are put into effect.
2. To appoint an Independent Data Monitoring Committee if appropriate
3. To monitor and supervise the progress of the DFEND trial, paying particular attention to recruitment, adherence to the protocol and patient safety.
4. To review at regular intervals relevant information from other sources (e.g. other related trials) and to recommend appropriate action (such as changes to the protocol, additional patient information, and stopping the study).
5. To review reports of serious adverse events or unexpected adverse reactions during the course of the trial.
6. To endeavour to ensure that the trial is conducted at all times to the rigorous standards set out in the MRC Guidelines for Good Clinical Practice and in line with EU legislation for clinical trials involving medicinal products.
7. To inform the sponsor and funding body on the progress of the DFEND trial
8. To advise the Trial Management Group on publicity and the presentation of all aspects of the DFEND trial.

12. Data Monitoring Committee

The terms of reference the Data Monitoring Committee are as follows:

- Periodically review and evaluate the accumulated study data for participant safety, study conduct and progress.
- Make recommendations to the TSC concerning the continuation, modification, or termination of the trial, ensuring that ethical considerations are of prime importance.
- To consider any requests for release of interim data and to recommend to the TSC on the advisability of this.
- In the event of further funding being required, to provide the TSC and the funding body appropriate information and advice on the data gathered to date that will not jeopardise the integrity of the study.

13. Direct Access to Source Data and Documents

Source notes document each contact (personal, telephone or written) with participants, their carers, family or associated health care professionals (e.g. Trust staff, GPs). They include the case report forms used to record the baseline assessments and follow-up assessments as well as basic details, eligibility assessments and logs of capacity assessments, consent, blood taking and transport, randomisation documentation, adverse events, unblinding and data queries. They are specific to the trial and will not be confused with case notes. Each entry into the source notes will be traceable, signed and dated. However participation/adverse event data in the trial will be documented in the patient's case notes. For participants recruited from PIC sites study participation/adverse event data will be relayed to the GP/Clinical team in writing or by secure encrypted email correspondence.

The intervention assignment will be kept securely by those with access to it and separately from the source notes and the main trial database until the trial is closed and / or un-blinded.

All personal information collected on DFEND trial participants and on potential participants will be treated as confidential information and will be handled according to guidelines set out in this document, associated SOP, and in accordance with the terms of the Data Protection Act 1998. All electronic files containing personal information will be held only on password-protected computers. The computers themselves will be kept securely. All other trial information such as the TMF, source notes and data keys will also be stored in a lockable facility and only accessed by delegated DFEND trial staff.

Access to personal information that is neither coded nor anonymised will be restricted to the smallest number of DFEND trial staff that will allow the study to be conducted effectively (this is expected to include the researchers, Trial Manager, Data Manager and Principal Investigator but may include other trial staff as necessary). Access to encoded or anonymised data will be under the control of the Principal Investigator/Data Manager, and will include sharing data periodically with the funder as part of the funding agreement.

Trial Identification Numbers will be used in all correspondence; names will not be used in any data transmissions or correspondence except in those to the patient's carer or clinicians.

In accordance with ICH-Good Clinical Practice (GCP) guidelines, the DFEND investigators will permit trial-related monitoring and quality assurance audits providing direct access to source data documents. A programme of internal monitoring will also be conducted by the Data Manager / Trial Manager throughout the course of the trial. The monitoring and data management processes are described in more detail within the study-specific data management plan and monitoring plan respectively. These have been created in compliance with King's Health Partners Clinical Trial Office (KHP-CTO) SOPs.

The source notes will be retained until at least 15 years after the last publication is released for this trial. The source notes will be kept separate from general medical notes to reduce the likelihood of the notes being destroyed in error.

14. Ethics & Regulatory Approvals

The trial will be conducted in compliance with the principles of the Declaration of Helsinki (1996), the principles of ICH-GCP and in accordance with all applicable regulatory requirements including but not limited to the Research Governance Framework and the Medicines for Human Use (Clinical Trial) Regulations 2004, as amended in 2006 and any subsequent amendments.

This protocol and related documents will be submitted for review to the Research Ethics Committee (REC), and to the Medicines and Healthcare products Regulatory Agency (MHRA) for Clinical Trial Authorisation.

The Chief Investigator will submit a final report at conclusion of the trial to the KHP-CTO (on behalf of the Sponsor), the REC and the MHRA within the defined timelines.

15. Quality Assurance

Monitoring of this trial will be to ensure compliance with Good Clinical Practice and scientific integrity will be managed and oversight retained, by the KHP-CTO Quality Team.

16. Data Handling

The Chief Investigator will act as custodian for the trial data. The following guidelines will be strictly adhered to:

- Patient data will be anonymised
- All anonymised data will be stored on a password protected computer.
- All trial data will be stored in line with the Medicines for Human Use (Clinical Trials) Amended Regulations 2006 and the Data Protection Act and archived in line with the Medicines for Human Use (Clinical Trials) Amended Regulations 2006 as defined in the Kings Health Partners Clinical Trials Office Archiving SOP.

The primary objective of good data handling and record keeping is to ensure that data collected on participants in the trial are accurate and complete and unbiased with respect to the trial intervention allocation.

Source data will be entered directly on to paper source data workbooks and then transferred to an electronic CRF via a customised Macro database created and maintained by the KCTU. The data entry screens will resemble the paper source document forms as closely as possible to minimise transcription error. All research staff will be advised to take care to ensure a thorough audit trail is maintained throughout the trial.

All participant data will be assigned a five digit patient identification number (PIN). The PIN will have no relation to the participants name or date of birth or any other patient identifier and will

be used on all study questionnaires and blood samples - no patient identifiable details will be recorded on the samples therefore the data gathered would not be identifiable to a third party and only the research team would be able to identify this data.

All study booklets will be stored in temperature managed, fireproof filing cabinets in access-controlled offices.

Access to the EDC (MACRO) database will be restricted to study researchers. Each researcher will have a personal username and password that only they know, thereby limiting unauthorised access to the electronic data, this will also allow for a detailed audit trail to be maintained within Macro.

17. Data Management

A data management plan will be written to detail central monitoring and data-integrity checking processes. All computers that will be used will have installed anti-virus software and staff will be informed of the importance of data protection and safe handling of data. There will be a manual and training slides provided to each end user to accompany the MACRO software to ensure each user is appropriately trained. The Macro development team will also be contactable if there are any issues with the database throughout the trial which will allow for proper database maintenance to occur.

As previously mentioned, all trial data will be collected on paper CRF and then transferred on to an electronic database. This will allow for a double-check of the data for good practice.

Flow Diagram of Data Handling Process:

Source data collected and entered onto paper source data workbooks

↓

Data transcribed from paper source data workbooks into Macro database via PC

↓

Data Collation, Queries, Verification & Cleaning via the MACRO database

↓

Data extracts for reports and analysis as required

↓

Data backed up and archived

18. Publication Policy

No report, either verbal or written, will be made without the prior approval of the Trial Steering Committee.

The trial team comprising all investigators, the trial manager, the trial statistician and members of the trial management group will be listed on all papers resulting from the main Statistical Analysis Strategy agreed by the TSC. Those contributing to the writing of papers arising from the Statistical Analysis Strategy will be included as co-authors on the relevant papers.

Independent members, other members and observers of the Trial Steering Group and the Data Monitoring and Ethics Committee (if appointed), will be acknowledged on all primary papers.

Research findings will be disseminated via peer-reviewed journals, conferences, internal reports and user group meetings. When the project is complete we will be able to provide all participants with a general summary of our research through a project newsletter. Our research study will also be described on the Institute of Psychiatry, Psychology and Neuroscience (IoPPN) general website (www.kcl.ac.uk), under the Department of Psychosis Studies.

19. Insurance / Indemnity

Arrangements for negligent harm and for compensation are covered by insurance and indemnity policies held by King's College London and the SLaM NHS Foundation Trust.

20. Financial Aspects

Funding to conduct the trial is provided by the Stanley Medical Research Institute (non-commercial).

21. Signatures

To be signed by Chief Investigator and statistician.

_____________________________________ __

Chief Investigator Date 25/07/2019

Print name: Fiona Gaughran

___ 7/8/2019

Statistician Date

Print name

22. DFEND protocol reference list:

1. DeLuca GC, Kimball SM, Kolasinski J, Ramagopalan SV, Ebers GC (2013) Review: the role of vitamin D in nervous system health and disease. Neuropathology and applied neurobiology. 39(5):458-484.
2. Eyles DW, Smith S, Kinove R, Hewison M, McGrath JJ (2005) Distribution of the vitamin D receptor and 1 alpha-hydroxylase in human brain. Journal of chemical Neuroanatomy. 29(1):21-30
3. Cui X, Pelekanos M, Liu PY, Burne THJ, McGrath JJ, Eyles DW (2013b) The vitamin D receptor in dopamine neurons; its presence in human substantia nigra and its ontogenesis in rat midbrain. Neuroscience. 236:77-87.
4. Puchacz E, Stumpf WE, Stachowiak EK, Stachowiak MK (1996) Vitamin D increases expression of the tyrosine hydroxylase gene in adrenal medullary cells. Brain research. Molecular Brain research. 36(1):193-6.
5. Groves NJ, Kesby JP, Eyles DW, McGrath JJ, Mackay-Sim A, Burne THJ (2013) Adult vitamin D deficiency leads to behavioural and brain neurochemical alterations in C57BL/6J and BALB/c mice. Behavioural Brain Research. 241:120-131
6. Brewer LD, Thibault V, Chen KC, Langub MC, Landfield PW, Porter NM (2001) Vitamin D hormone confers neuroprotection in parallel with downregulation of L-type calcium channel expression in hippocampal neurons. The journal of Neuroscience. 21(1):98-108.
7. Torrey EF, Miller J, Rawlings R, Yolken RH (1997) Seasonality of births in schizophrenia and bipolar disorder: a review of the literature. Schizophrenia research. 28(1):1-38.
8. Davies G, Welham J, Chant D, Torrey EF, McGrath J (2003) A systematic review and meta-analysis of northern hemisphere season of birth studies in schizophrenia. Schizophrenia Bulletin. 29(3):587-593.
9. Torrey EF (1987) Prevalence studies in schizophrenia. British Journal of Psychiatry. 150:598-608.
10. Cantor-Graae E and Selten JP (2005) Schizophrenia and migration: a meta-analysis and review. American Journal of Psychiatry. 162:12-24.
11. McGrath JJ, Burne TH, Feron F, Mackay-Sim A, Eyles DW (2010a) Development vitamin D deficiency and risk of schizophrenia: a 10-year update. Schizophrenia Bulletin. 36(6)1073-8.
12. McGrath JJ, Eyles DW, Pedersen CB, Anderson C, Ko P, Burne TH, Norgaard-Pedersen B, Hougaard DM, Mortensen PB (2010b) Neonatal vitamin D status and risk of schizophrenia: a population-based case-control study. Archive of General Psychiatry. 67(9):889-94.
13. Tolppanen AM, Sayers A, Fraser WD, Lewis G, Zammit S, McGrath J, Lawlor DA (2012) Serum 25-hydroxyvitamin D3 and D2 and non-clinical psychotic experience in childhood. PLoS One. 7(7):e41575.
14. Hedelin M, Lof M, Olsson M, Lewander T, Nilsson B, Hultman CM, Weiderpass E (2010) Dietary intake of fish, omega-3, omega-6 polyunsaturated fatty acids and vitamin D and the prevalence of psychotic-like symptoms in a cohort of 33 000 women from the general population. BMC Psychiatry. 10:38
15. McCann and Ames (2008) Is there convincing biological or behavioural evidence linking vitamin D deficiency to brain dysfunction? The FASEB Journal. 22(4):982-1001.

16. Feron F, Burne THJ, Brown J, Smith E, McGrath JJ, Mackway-Sim A, Eyles DW (2005) Developmental Vitamin D3 deficiency alters the adult rat brain. Brain research Bulletin. 65(2):141-148
17. Balden R, Selvamani A, Sohrabji F (2012) Vitamin D deficiencies exacerbates experimental stroke injury and dysregulates Ischemia-induces inflammation in adult rats. Endocrinology. 153(3):2420-35.
18. Suzuki M, Yoshioka M, Hashimoto M, Murakami M, Noya M, Takahashi D, Urashima M (2013) Randomised, double-blind, placebo-controlled trial of vitamin D supplementation in Parkinson's disease. American Journal of clinical nutrition. 97(5):1004-13.
19. Belvederi Murri M, Respino M, Masotti M, Innamorati M, Mondelli V, Pariante C, Amore M (2013) Vitamin D and psychosis: mini meta-analysis. Schizophrenia research. 150(1):235-9
20. Crews M, Lally J, Gardner-Sood P, Howes O, Bonaccorso S, Smith S, Murray RM, Di Forti M, Gaughran F (2013) Vitamin D deficiency in first episode psychosis: as case-control study. Schizophrenia Research. 150(2-3):533-7
21. Graham KA, Keefe RS, Lieberman JA, Calikoglu AS, Lansing KM, Perkins DO (2014) Relationship of low vitamin D status with positive, negative and cognitive symptom domains in people with first-episode schizophrenia. Early Intervention in Psychiatry. 9(5):397-405.
22. Tiangga E, Gowda A, Dent JA (2008) Vitamin D deficiency in psychiatric in-patient and treatment with daily supplements of calcium and ergocalciferol. Psychiatric Bulletin. 32(10):390-393.
23. Dealberto MJ (2013) Clinical symptoms of psychotic episodes and 25-hydorxy vitamin D serum levels in black first-generation immigrants. Acta psychiatrica Scandinavica. 128(6):475-87.
24. Thakurathi N, Stock S, Oppenheim CE, Borba CP, Vincenzi B, Seidman LJ, Stone WS, Henderson DC (2013) Open-label pilot study on vitamin D3 supplementation for antipsychotic-associated metabolic anomalies. International clinical psychopharmacology. 28(5):275-82.
25. Amminger GP, Schafer MR, Papageorgiou K, Klier CM, Cotton SM, Harrigan SM, Mackinnon A, McGorry PD, Berger GE (2010) Long-chain omega-3 fatty acids for indicated prevention of psychotic disorders: a randomized, placebo controlled trial. Archive of General psychiatry. 67(2):146-54.
26. Leucht S, Burkard T, Henderson J, Maj M, Sartorius N (2007) Physical illness and schizophrenia: a review of the literature. Acta psychiatrica Scandinavica. 116(5):317-333.
27. Berk M, Jacka FN, Williams LJ, Ng F, Dodd S, Pasco JA (2008) Is this D vitamin to worry about? Vitamin D insufficiency in an inpatient sample. Australian & New Zealand Journal of Psychiatry 42(10):874-878.
28. Kishimoto T, DeHert M, Carlson HE, Manu P, Correll CU (2012) Osteoporosis and fracture risk in people with schizophrenia. Current opinion in Psychiatry. 25(5):415-429.
29. Partti K, Heliovaara M, Impivaara O, Perala J, Saarni SI, Lonngvist J, Suvisaari JM (2010) Skeletal status in psychotic disorders: a population-based study. Psychosomatic medicine. 72(9):933-40
30. Meaney AM and O'Keane V (2007) Bone mineral density changes over a year in young females with schizophrenia: Relationship to medication and endocrine variables. Schizophrenia Research. 93(1-3):136-143.

31. Hypponen E, Boucher BJ, Berry DJ, Power C (2008) 25-hydroxyvitamin D, IGF-1, and metabolic syndrome at 45 years of age: a cross-sectional study in the 1958 British Birth Cohort. Diabetes. 57(2):298-305.
32. Melamed ML, Michos ED, Post W, Astor B (2008) 25-hydroxyvitamin D levels and the risk of mortality in the general population. Archives of Internal Medicine. 168(15):1629-37.
33. Plum LA and DeLuca HF (2010) Vitamin D, disease and therapeutic opportunities. Nature Reviews Drug Discovery. 9:941-955.
34. Kupferschmidt (2012) Uncertain verdict as vitamin D goes on trial. Science. 337(6101):1476-8

Randomised, double-blind, placebo-controlled, parallel-group trial of Vitamin D supplementation compared to placebo in people presenting with their First Episode of psychosis Neuroprotection Design (DFEND)

EudraCt Number: 2014-002639-32

Statistical Analysis Plan
Version 1.10
Version 1.10 10/04/2019

Trial Statistician: Dominic Stringer

Signature.............................. Date 29/05/2019

Chief Investigator: Dr Fiona Gaughran

Signature.............................. Date 30/05/2019

Trial Steering Committee Chair: Professor David Osborn

Signature.............................. Date 22/5/19

Purpose and Scope of Statistical Analysis Strategy

This document details the presentation and analysis strategy for the primary papers reporting results from the DFEND trial. It is intended that the results reported in these papers will follow the strategy set out herein; subsequent papers of a more exploratory nature will not be bound by this strategy but will be expected to follow the broad principles laid down for the principal paper(s). These principles are not intended to curtail exploratory analysis or to prohibit sensible statistical and reporting practices, but they are intended to establish the strategy that will be followed as closely as possible, when analysing and reporting the trial. Reference was made to the trial protocol (V9.2 01.10.2018), ICH [1] guidelines on Statistical Principles (E9) and CONSORT [2] guidelines.

This document contains up to date statistical analysis plans (with version numbers and dates).

A) Quantitative Analysis Plan
B) Schedule of Assessments and Measures

Contents

A) QUANTITATIVE ANALYSIS PLAN 4

1. Description of the trial 5

1.1 Principal research objectives to be addressed 5
1.2 Trial design including blinding 5
1.3 Method of allocation of groups 8
1.4 Duration of the treatment period 8
1.5 Frequency and duration of follow-up 8
1.6 Visit windows 8
1.7 Data collection 8
1.8 Sample size estimation (including clinical significance) 10
1.9 Brief description of proposed analyses 11

2. Data analysis plan – Data description 12

2.1 Recruitment and representativeness of recruited patients 12
2.2 Baseline comparability of randomised groups 14
2.3 Characteristics of baseline vitamin D 14
2.4 Adherence to allocated treatment and treatment fidelity 14
2.5 Loss to follow-up and other missing data 14

3. Data analysis plan – Inferential analysis 16

3.1 Main analysis of treatment differences 16
3.1.1 Analysis of primary outcomes 16
3.1.2 Analysis of secondary outcomes 17
3.1.3 Statistical considerations 18
3.1.4 Sensitivity analyses 20
3.1.5 Planned subgroup analyses 20
3.2 Exploratory moderator analysis 20
3.3 Interim analysis 20

4. Software 20

B) SCHEDULE OF ASSESSMENTS AND MEASURES 21

C) REFERENCE LIST 23

D) AMENDMENTS 23

A) QUANTITATIVE ANALYSIS PLAN

Investigators
Prof John McGrath
Dr Shubulade Smith
Dr Michael Berk
Prof David Taylor
Prof Philip McGuire
Prof Sabine Landau

Principal investigator
Dr Fiona Gaughran

Trial manager
Dr Gabriella Wojewodka

Trial statistician
Professor Sabine Landau
Dominic Stringer

1. Description of the trial

1.1 Principal research objectives to be addressed

We hypothesise that optimal vitamin D status could be neuroprotective in those with first episode psychosis (FEP).

Primary objectives

- To determine whether the addition of 120,000 IU monthly of vitamin D (cholecalciferol) supplement to standard treatments is more efficacious than placebo in improving outcomes (Positive and Negative Syndrome Scale (PANSS) total score) at 6-months post randomisation follow up in those with First Episode Psychosis.

Secondary objectives

To examine the following broader range of clinically-relevant outcomes:

- PANSS total score at 3 months
- PANSS subscores at 3 and 6 months:
    - Positive Scale
    - Negative Scale
    - General Psychopathology Scale
- Global Assessment of Function (GAF) at 6 months
- Calgary Depression Scale (CDS) at 6 months
- Cardiovascular risk markers at 6 months:
    - Waist circumference (cm)
    - BMI (kg/m^2)
    - HbA1c (mmol/mol)
    - Total Cholesterol (mmol/L)
    - CRP (mg/L)
- 25-hydroxyvitamin D (25OHD) Concentrations at 6 months

We will examine these outcomes in (a) all participants and (b) in a subgroup of patients with suboptimal vitamin D concentrations at baseline (except for 25OHD blood concentration levels).

1.2 Trial design including blinding

We propose a randomised, double-blind, placebo-controlled, parallel-group trial of Vitamin D supplementation compared to placebo in people presenting with their first episode of psychosis. Study participants will be on active study treatment for a maximum of 6 months.

The trial will be double blind. Active and placebo oral medication will be produced and packaged identically. Clinicians and research workers completing baseline, clinic attendance assessments and all follow-ups will be blind to group allocation, as will patients and pharmacists. This design will ensure that the study has a high level of both treatment integrity

(delivery of the treatment as intended) and treatment differentiation (treatment conditions differed from one another in the intended manner). The trial has two groups:

- Group A: 50% of participants will be randomised to Active Treatment with Cholecalciferol (vitamin D3), 120,000 IU per month (equivalent to 4,000 IU per day), given orally as Vigantol® oil by Merck Sereno GmbH, Germany (cholecalciferol dispersed in triglyceride oil as vehicle; 8 ml bottles, containing 20,000 IU cholecalciferol per 1ml drop, and administered as 6mL given in a graduated oral syringe by a fully trained member of the research team).
- Group B: For the 50% receiving placebo, we will use identical bottles containing an organoleptically matched triglyceride oil (Miglyol® 812 oil). Administration of the placebo will be identical to the active treatment.

Figure 1. Trial design flow diagram

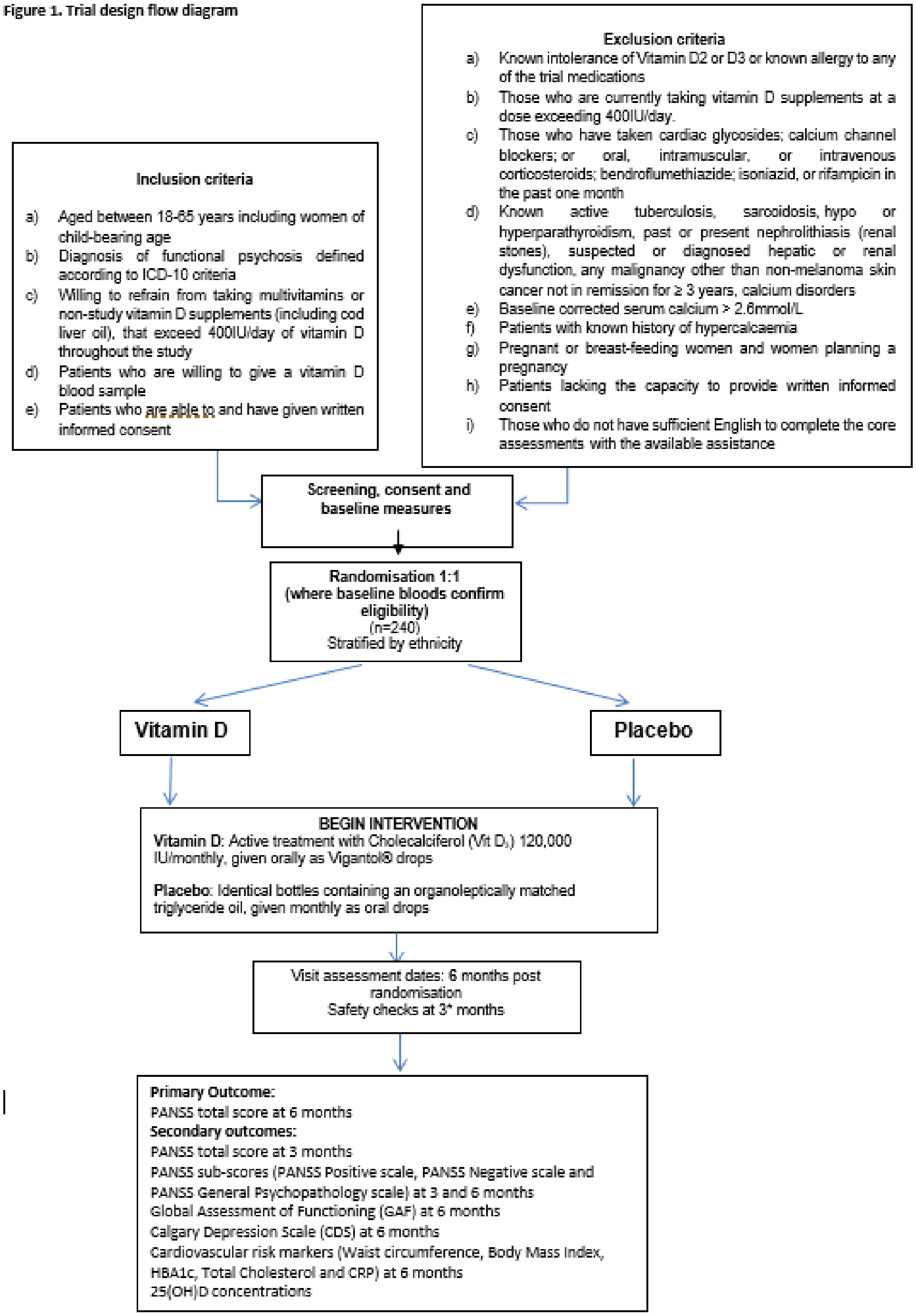

1.3 Method of allocation of groups

An online randomisation service will be provided by the King's Clinical Trials Unit. Randomisation will take place after collection of baseline measures. The unit of randomisation will be the individual participant. Randomisation will be at a 1:1 ratio and the method of randomisation will be stratified randomisation with randomly varying block sizes of 2-4 where the stratification factor is ethnicity (with 2 levels: White, Other).

We chose to balance treatment arms by ethnicity as this is thought to predict outcomes. There are no known predictors of PANSS in our target population. However, blood vitamin D concentration varies by ethnicity; and we hypothesize that blood vitamin D concentration mediates any effect of vitamin supplement. Thus, ethnicity was chosen as a randomisation stratifier.

1.4 Duration of the treatment period

Participants will receive a monthly dose of either Vigantol Oil 120,000 IU per month (equivalent to 4,000 IU per day) or placebo.

The end of the trial will be defined as the last patient's post 6-month visit telephone call. Each participant will remain on the trial until they have each completed the post 6-month visit telephone call. The study will run from December 2015 until December 2019 to ensure that each participant has 6 months of exposure to either the active or placebo treatment.

1.5 Frequency and duration of follow-up

Primary and secondary measures will be taken at baseline (pre-randomisation), and 6 months post-randomisation, there will be an additional PANSS measure at 3 months post-randomisation. Safety outcomes will be additionally collected at 3 months post-randomisation.

1.6 Visit windows

For the follow-up assessment at 6 months a visit window of -4/+6 weeks will be set to ensure follow-up data is collected. For routine safety blood visits, a -2 week window will be adhered to, but an upper time frame will not be specified for collecting the safety bloods as these must be performed.

1.7 Data collection

<u>Eligibility screening</u>

The study population will be patients in their first episode of psychosis (FEP) defined as first presentation to services within the last three years.

<u>Inclusion Criteria</u>

All patients within psychosis services, including Early Intervention Services (EIS) and First Episode Psychosis (FEP) inpatient units who meet the following criteria will be invited to participate in the study:

1. Aged between 18-65 years old including women of child-bearing age
2. Having a diagnosis of functional psychosis FEP defined according to ICD-10 criteria for psychosis (codes F20-29 and F30-33).
3. Willing to agree to refrain from taking multivitamin or non-study vitamin D supplements that exceed 400IU/day throughout the study

4. Willing to give a blood vitamin D sample at baseline
5. Patients who are able and have given written informed consent.

Exclusion Criteria

1. Known intolerance of Vitamin D2 or D3 or known allergy to any of the trial medications
2. Those who are currently taking vitamin D supplements at a dose exceeding 400IU/day
3. Those who have taken cardiac glycosides, calcium channel blockers or oral, intramuscular or intravenous corticosteroids, bendroflumethiazide, isoniazid and rifampicin in the past one month
4. Known active tuberculosis, sarcoidosis, hypo or hyperparathyroidism, past or present nephrolithiasis (renal stones), suspected or diagnosed hepatic or renal dysfunction, any malignancy other than non-melanoma skin cancer not in remission for ≥ 3 years, calcium disorders
5. Baseline corrected serum calcium > 2.6 mmol/L
6. Patients with known history of hypercalcaemia
7. Pregnant or breast-feeding women and women planning a pregnancy
8. Patients lacking the capacity to provide written informed consent
9. Those who do not have sufficient English to complete the core assessments with the available assistance

Measures
A detailed description of data collected will be given in the Schedule of Assessments and Measures (section B of this document). What follows is a brief overview to aid understanding of the analysis plan.

Baseline
- Eligibility and consent
- Socio-demographics
- Duration of untreated psychosis
- Current medication
- Vitamin supplementation
- Anthropometrics
- Blood sampling full blood count
- Urine pregnancy test
- Smoking history
- Sun exposure measure
- IPAQ
- Fitzpatrick Skin Type Questionnaire
- Vitamin D food questionnaire
- Blood sampling for calcium levels

Primary outcome measures at baseline, 3 months and 6 months post randomisation
- PANSS Total Score

Secondary outcome measures at baseline, 3 months and 6 months post randomisation
- PANSS Positive scale sub-score
- PANSS Negative scale sub-score

- PANSS General Psychopathology sub-score

Secondary outcome measures at baseline and 6 months post randomisation

- GAF score
- CDS score
- Waist circumference (cm)
- BMI (kg/m^2)
- HbA1c (mmol/mol)
- Total Cholesterol (mmol/L)
- CRP (mg/L)
- Blood sampling vitamin D concentrations

Mediators of treatment

Vitamin D concentrations may be a mediator of treatment effect on PANSS. If Vitamin D supplementation increases blood concentrations, we could demonstrate that any change in PANSS is due to changes in blood concentrations.

Putative Moderators of treatment

Cardiovascular and inflammatory markers

Adverse events

- Reported adverse events

Additional post-randomisation (follow-up) measures

- Concomitant medications
- Withdrawals from follow up
- Treatment guess
- Dosing regime

1.8 Sample size estimation (including clinical significance)

Sample size

A total of 240 first episode psychosis patients will be recruited over a two-year period.

Power calculation

Based on recently collected PANSS Total score in FEP patients collected as part of the Physical Health and Substance Use Measures in first episode Psychosis (PUMP) study (n = 190), the mean (and standard deviation) score was 58.1 (15.0) units. Based on the number of new cases presenting to FEP services and based on the annual counts of FEP patients recruited into similar studies, we predict that we will recruit 120 patients per year (i.e. 240 over two years).

The primary intention to treat (ITT) analyses will compare two equally sized treatment arms, treatment or placebo on the PANSS scale at 6 months post-randomisation. For the power analyses, we modelled two plausible scenarios. For the primary outcome (six-month follow-up), we assumed a 20% attrition proportion. After 20% attrition the sample size is 192, 96 in each trial arm).

Based on alpha = 0.05 and power = 80%, samples between 200 and 180 participants will be able to confidently detect mean PANSS total score group differences of between 6 and 6.3 units This equates to a standardised effect of size of approximately 0.4 to 0.42. Considering

90% power with the same power assumptions, we will be able to detect mean PANSS total score group differences of between units 6.9 and 7.3 units (standardised effect size from 0.46 to 0.49).
We also wish to estimate the average treatment effect in those participants who meet the vitamin D threshold. We assume 60% of the FEP have vitamin D concentrations below 50 nmol/L. At 6 months after 20% attrition (sample now = 192) and 60% who meet the vitamin D threshold, this will result in a projected sample size of 115.
Based on alpha = 0.05 and power = 80%, samples between 120 and 100 participants will be able to confidently detect mean PANSS total score group differences of between 7.7 and 8.5 units This equates to a standardised effect of size of approximately 0.52-0.57. Considering 90% power with the same power assumptions, we will be able to detect mean PANSS total score group differences of between 8.95 and 9.8 units (standardised effect size from 0.60 to 0.65).

1.9 Brief description of proposed analyses

Analyses will be carried out by the trial statistician. The trial statistician will remain blind until all analyses are completed. The analysis of the secondary outcome of blood sampling vitamin D concentrations at 6 months will be carried out after all other analyses are completed as there is a high expectation that mean concentration will be higher in the intervention group.

The primary analyses of efficacy will be based on the intention-to-treat sample, utilising all available follow-up data from all randomised participants. The significance level will be 5% (2-sided) for all specified main and secondary analyses with estimates and confidence intervals presented for all effects.

All descriptive analyses, recruitment rate, consent rate, loss to follow-up, departures from randomised treatment and the prevalence of serious adverse events will be reported post-randomisation and summarised by treatment arm over the course of the study. All causes of withdrawal from randomised treatment will be reported.

The main objective of the statistical analyses is to assess the effect of vitamin D supplementation on the primary outcome PANSS total Score at the 6-month follow-up time point. To this end linear mixed modelling (LMM) will be employed.

Treatment effects on secondary outcomes will be using generalized linear models allowing for non-normal data where necessary. Treatment arm, baseline measurement of the outcome and randomization stratifier (ethnicity) will be included as covariates.

Average treatment effects (ATE) with the FEP population will be estimated at 3 and 6 months for the primary outcome and at 6 months for all secondary. We will also estimate subpopulation (baseline vitamin D insufficient and sufficient) specific treatment effects for the primary outcome and all secondary outcomes, except for 25OHD blood concentration levels, by use of an interaction of baseline vitamin D insufficiency status with treatment group in the statistical models.

We expect there to be some missing data in the post-treatment outcomes variables. The LMM analyses are based on maximum likelihood and will provide valid inferences under a missing at random (MAR) missingness mechanism. We will explore predictors of missingness, if deemed suitable for adjustment we will include these as explanatory

variables in the analyses. If post randomisation or unsuitable baseline variables are identified a Multiple Imputation [3] model will be considered instead.

Sensitivity analyses will be used to assess the robustness of conclusions to data missing not at random (MNAR) outcome data and to departures from randomised treatment in the manner of White and colleagues [4].

Data summaries and analyses will be carried out in Stata.

2. Data analysis plan – Data description

2.1 Recruitment and representativeness of recruited patients

A CONSORT flow chart will be constructed [2]– see Figure 2. This will include the number of eligible patients, number of patients agreeing to enter the trial, number of patients refusing, then by treatment arm: the number of patients not / inadequately / adequately treated OR compliant / non-compliant, the number continuing through the trial, the number withdrawing, the number lost to follow-up and the numbers excluded / analysed.

Figure 2. Template CONSORT diagram for DFEND trial

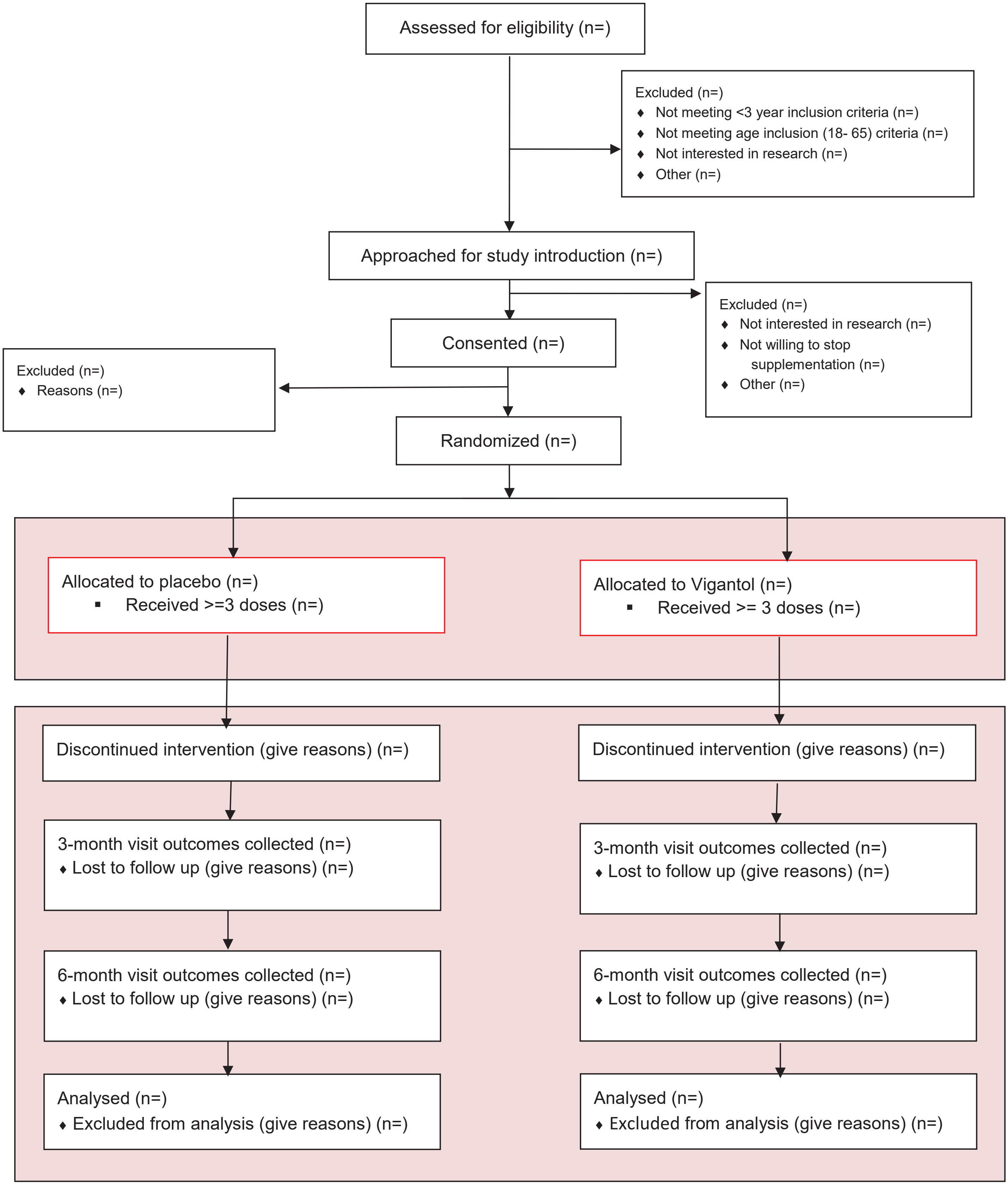

2.2 Baseline comparability of randomised groups

Baseline descriptions of participants by treatment and overall: means and standard deviation, medians and upper and lower quartiles, or numbers and proportions will be reported as appropriate.

No significance testing will be used to test baseline difference between the randomised treatment groups [5].

The following baseline variables will be described both overall and between randomised groups:

1. Stratifications factors (ethnicity) and sociodemographics

2. Biomedical status will be measured as: waist circumference, weight, height (for BMI), blood pressure, heart rate (pulse), along with blood measures of cardiovascular risk / inflammatory markers and smoking habits.

3. Mental health status: The Positive and Negative Syndrome Scale (PANSS); Global Assessment of Functioning (GAF); Calgary Depression Scale (CDS).

4. Levels of sun exposure, including type of clothing worn, levels of physical activity (IPAQ), skin type (Fitzpatrick Skin Type questionnaire), level of vitamin D supplementation, vitamin D food frequency questionnaire.

2.3 Characteristics of baseline vitamin D

Baseline descriptions of levels of sun exposure and vitamin D supplementation will be summarised by baseline vitamin D level (sufficient vs insufficient as defined by baseline blood vitamin D concentration levels greater or less than 50 nmol/L). The association between these variables and blood vitamin D concentrations can be assessed using suitable statistical tests (chi—squared, Fisher's Exact or T test).

- Levels of sun exposure, including type of clothing worn, levels of physical activity (IPAQ), skin type (Fitzpatrick Skin Type questionnaire), vitamin D food frequency questionnaire, level of vitamin D supplementation, season of randomisation.

2.4 Adherence to allocated treatment and treatment fidelity

Adherence to the oral study medication can be ascertained via the dosing schedule.

The numbers and proportions of participants in each of the treatment arms who were given the treatment that they were randomised to will be described. Adherence, including number of doses received will also be described.

2.5 Loss to follow-up and other missing data

It is the aim of the trial to minimise withdrawal of participants from treatment and follow-up. Completion of a withdrawal from trial is regarded as withdrawal from follow-up in this analysis.

Withdrawal from trial will be reported by intervention group. Moreover, the proportions of participants missing each variable will be summarised in each arm and at each time point.

The numbers, proportions and reasons for withdrawal from trial will be summarised by treatment arm. The distribution of times between randomisation and withdrawal from follow-up will be summarised using a histogram.

The baseline characteristics of those missing follow-up will be compared to those with complete follow-up. The relationship between baseline characteristics and missing data will be investigated graphically. Missing data patterns will be examined using a logistic predictor of missingness model.

2.6 Adverse event reporting

Adverse events (AE), adverse reactions (AR), serious adverse events (SAE) and serious adverse reactions (SAR) will be summarised by intervention arm and time point.

2.7 Assessment of outcome measures (unblinding)

The outcome assessors, trial statistician and all members of the study team will be blinded. The data monitoring committee (DMC) may be unblinded at their request, and if they are unblinded, will be reminded not to unblind the trial statistician or other members of the study team at the beginning of every meeting.

2.8 Descriptive statistics for outcome measures

Each of the outcome measures will be described by treatment group. Means and standard deviations or medians and interquartile ranges will be used for continuous variables; Q-Q plots will be used to assess whether the distribution of a variable is normal. Frequencies and proportions will be used to describe categorical variables.

3. Data analysis plan – Inferential analysis

3.1 Main analysis of treatment differences

The main statistical analyses will estimate the difference in mean outcomes between patients randomised to the vitamin supplementation and placebo by intention to treat at 6 months' post randomisation on the PANSS. ATE estimates will be summarized at 3, 6 months for the primary outcome and at 6 months for secondary outcomes. We will also estimate subpopulation (baseline vitamin D insufficient and sufficient) specific treatment effects for all statistical models.

Missing post randomisation assessments will be dealt with by fitting adequate linear models to all the variables using maximum likelihood methods. Such an approach provides valid inferences under the assumption that the missing data mechanism is ignorable (MAR) and if predictors of missingness are included as covariates in the model.

The significance level will be 5% (two-sided) for specified analyses. Group difference estimates and associated 95% confidence intervals will be reported.

The trial statistician will remain blind until the main analyses have been completed. The analysis of the secondary outcome of vitamin D concentrations at 6 months will be carried out after all other analyses are completed as there is a high expectation that mean concentration will be higher in the intervention group.

Sensitivity analyses will be used to assess the robustness of conclusions to missing outcome data and to departures from randomised treatment.

3.1.1 Analysis of primary outcomes

Primary Efficacy Parameters

A. To determine whether the addition of 120,000 IU monthly of vitamin D supplement to standard treatments is more effective than placebo in improving PANSS total score at six-month follow-up in those with First Episode Psychosis

The main objective of the statistical analyses is to assess the effect of vitamin D supplementation on the primary outcome PANSS total score at the 6-month follow-up time point. To this end linear mixed modelling (LMM) will be employed.

Linear mixed modelling utilises all available information, leading to more precise estimates of the treatment effect at the time points. This technique will allow the simultaneous modelling of the repeated outcome time points, description of the course of the outcomes over time in the two intervention groups and evaluate the resulting differences between the groups.

Prior to modelling the longitudinal effect of the intervention groups over the time course of the study, spaghetti plots will be used to examine patterns of the outcome over time.

The total scores are assumed to arise from a normal distribution. This will be checked and if this is found not to be appropriate, transformations may be used.

In such models the outcome variable measured at the post treatment time points (here 3 and 6 months) features as the dependent variable with, with treatment arm (vitamin D or placebo), time (3 or 6 months), time by treatment arm interaction, baseline PANSS and the randomization stratifier (ethnicity) included as covariates.

In this longitudinal analysis, each participant acts as a cluster. Therefore, for the random part of the model, random intercepts that vary at the participant level will be used.

The Stata command 'mixed' will be used to fit the model. Treatment differences will be estimated for each time point separately using the 'lincom' command, enabling the outcome at the primary endpoint to be found this way.

Planned Subgroup Analysis

B. To determine whether the addition of 120,000 IU monthly of vitamin D supplement to standard treatments is more effective than placebo in improving PANSS total score at six-month follow-up in those with First Episode Psychosis who have suboptimal vitamin D concentrations at baseline (defined as less than 50 nmol/L).

To estimate treatment effects in the planned subgroup analyses we will include a treatment x time x baseline vitamin D insufficiency status interaction term (and all lower order interaction terms) as covariates in the modelling process.

As mentioned previously, we will produce estimates of ATE (relating to whole FEP target population) as well as estimates of average treatment effects within the vitamin D deficient (not deficient) subpopulations.

We will also estimate treatment effects in this subgroup (using an interaction term in the models) for all secondary outcomes except for 25OHD blood concentration levels.

3.1.2 Analysis of secondary outcomes

Secondary Efficacy Parameters

Treatment effects on all secondary outcomes that were measured repeatedly over the follow-up period will lend themselves to the analysis described above. These secondary outcomes will be assessed using similar modelling techniques, employing generalisations to non-normal data where necessary or transformation of the outcome variable. Where secondary outcomes are only measured once over the follow up period, generalised linear models will be used.

1. Positive and Negative Syndrome Scale (PANSS) at 3 months
2. PANSS Positive Scale subscore at 3 and 6 months
3. PANSS Negative Scale subscore at 3 and 6 months
4. PANSS General Psychopathology Scale subscore at 3 and 6 months
5. Global Assessment of Functioning (GAF) at 6 months
6. Calgary Depression Scale (CDS) at 6 months
7. Waist circumference (cm) at 6 months
8. BMI (kg/m^2) at 6 months
9. Total Cholesterol (mmol/L) at 6 months
10. HbA1c (mmol/mol) at 6 months

11. CRP (mg/L) at 6 months
12. Efficacy of vitamin D supplementation at 6 months indicated by 25OHD blood concentration level

Safety of Vitamin D

These analyses are based in the safety data and describe safety outcomes. Chi squared tests (Fisher's Exact test where expected count in any cell is less than 5) will be used to describe any differences. SAEs will be defined in accordance with the EU clinical trial directives. The potential adverse events that we have anticipated we will summarise separately.

AEs will be tabulated by event type and clinical classification by time, phase and randomised group. Each table will detail the number of participants that were still in the trial at the time points by randomisation group. If any adverse events are selected as being of interest they will be further summarised; outlining the severity (mild, moderate, severe) and if classified serious, the expectedness (expected, unexpected) of the event. Any death will be reported as a serious adverse event.

Mechanism of action

The study is based on the theoretical model that vitamin D supplementation might improve psychotic symptoms (PANSS) by increasing blood vitamin D levels to sufficient levels. We wish to investigate this empirically by partitioning any detected vitamin D supplement effect on PANSS into two components: (a) A part that is mediated via changing blood concentration levels (indirect effect) and (b) a non-mediated part (direct effect). To this end we will employ appropriate mediation methods such as the product of coefficients method or linear structural equation models. These methods rely on the assumption that all confounders of the blood level – PANSS path have been measured and included in the models [6].

Inflammatory markers

Exploratory moderator analyses not considered for the primary publication, please see section 3.2.

3.1.3 Statistical considerations

Time points

This analysis will use data from all available time points. Deviations of measurements from planned time points will be summarised by treatment group.

Stratification and clustering

Randomisation has been stratified by ethnicity (two level categorical variable; white or other). Therefore, it is important to include this variable in the modelling process. The level of the stratification factor that has the largest number of participants will be used as the reference category in the modelling.

The structure of primary outcome (and some of the secondary outcome) data is longitudinal with repeated measurements. This correlation of observations within participants will be accounted for in the modelling process by including a subject-varying random intercept.

Missing items in scales and subscales

The number (%) with complete data will be reported. The ideal approach would be to use missing value guidance provided for scales, if provided by the authors. As an alternative, scales will be pro-rated for an individual if 20% or fewer items are missing. For example, in a scale with 10 items, prorating will be applied to individuals with 1 or 2 items missing. The average value for the 8 or 9 complete items will be calculated for that individual and used to replace the missing values. The scale score will be calculated based on the complete values and these replacements.

Missing baseline data

Missing baseline data should not be an issue for the primary analysis. Some extensions to this analysis may use other baseline variables; if these contain missing data, the number with complete data will be reported and they will be imputed using a method suitable (single round of multiple imputation) to the variable as per the recommendations of White et al [7] .

Missing outcome data

The mixed model will be fitted using maximum likelihood methods that are valid under the MAR assumption. However, this assumption relates to the variables that are included in the model. To allow for a variable which predicts missingness, this variable needs to be included as either one of the explanatory or dependent variables in the model. Should the investigation into missingness indicate any demographic or clinical baseline variables that are predictors of outcome missingness then such variables will be included as covariates in the model if deemed suitable for adjustment.

If post treatment variables such as compliance with treatment, or baseline variables not suitable to adjust for in the main analyses, are found to be predictive of drop out, multiple imputation will be considered.

Method for handling multiple comparisons

No adjustment will be considered for multiple comparisons, allowing reviewers to make their own adjustment to estimates if they wish [8].

Method for handling non-compliance

Compliance is defined as receiving at least 3 doses prior to the primary endpoint (6 months post-randomisation). This will be summarised and if there is considerable non-compliance, we will look at estimating efficacy using a complier average causal effect (CACE) analysis.

Model assumption checks

The models assume normally distributed outcomes; this will have been checked when describing the data and if substantial departures from normality occur, transformations will be considered. Residuals will be plotted to check for normality and inspected for outliers. Bootstrapping of the standard errors (and subsequent confidence intervals) may be considered where residuals appear non-normal.

3.1.4 Sensitivity analyses

The impact of departures from MAR on treatment effects will be assessed using sensitivity analysis.
The impact of any changes to the inclusion/exclusion criteria during the course of the trial will also be assessed using a sensitivity analysis/analyses.

3.1.5 Planned subgroup analyses

None other than outlined above.

3.2 Exploratory moderator analysis

The inflammatory markers will not form part of the primary analyses. These post hoc analyses will only become clear once the main objectives of the trial have been evaluated. The trial population for which we wish to investigate these markers as potential predictor markers (predictors of treatment effects) can only be defined once the investigators have a clear summary of where vitamin D is effective, i.e. in the entire (non-tested) population or the vitamin D insufficient / sufficient population. Interaction with treatment effects can be used to evaluate these variables.

3.3 Interim analysis

No interim analyses are planned for this trial.

4. Software

Data management: An online data collection system for clinical trials (MACRO; Elsevier) will be used. This is hosted on a dedicated server at KCL and managed by the King's CTU. The KCTU will extract data periodically as needed and provide these in comma separated (.csv) or Stata (.dta) format.

Statistical analysis: Stata 15.1 will be used for the main analyses. R may additionally be used for production of graphs and for automating report writing.

B) SCHEDULE OF ASSESSMENTS AND MEASURES

Data being collected from patients and recorded in MACRO:

STUDY PERIOD	SCREENING AND BASELINE	RANDOMISATION	TREATMENT Vigantol®/Placebo	SAFETY CHECK	ASSESSMENT VISIT	FOLLOW UP PHONE CALL
**VISIT Name**	**Baseline VISIT**		**DOSING VISITS 1 – 6**	**3 MONTH VISIT**	**6 MONTH VISIT**	**POST 6 month CONTACT**
**TIMEFRAME**	**WEEK -2**	**WEEK 0**	**Monthly for 6 months (Visit 1 is the anchor date, Visits 2 to 6: -2/+2 weeks from anchor date)**	**MONTH 3**	**MONTH 6 (Visit 6 assessment: -4/+6 weeks from anchor date)**	**POST 6 mth**
Eligibility & Informed consent	X					
Sociodemographics	X					
NOS (Duration of Untreated Psychosis)	X					
Current medication	X		X	X	X	
Medical history	X					
Vitamin supplementation	X		X	X	X	
Anthropometrics	X				X	
Blood sampling: vitamin D concentration (storage)	X				X	
Blood sampling: incl. clinical, genetic, cardiovascular and inflammatory markers	X				X	
Urine pregnancy test	X*		X*	X*	X*	
Smoking habits	X				X	
PANSS	X			X	X	
GAF	X				X	
CDS	X				X	
OPCRIT	X					
Sun Exposure questionnaire	X				X	
IPAQ	X				X	
SIMPAQ	X				X	
Fitzpatrick Skin Type Questionnaire	X					
Vitamin D Food Frequency Questionnaire	X				X	

Randomisation		X				
Adverse events recorded			X	X	X	X
Blood sampling for calcium levels (including parathyroid hormone test if Calcium is abnormal)	X		X**	X	X	
Administration of IMP or Placebo			X***	X***	X***	
Patient Medication Guess			X	X	X	X
Service Contacts Form	X				X	
Patient Medication Guess Form			X	X	X	X

* In the event that it is not possible to obtain a urine sample, a blood HCG sample will be obtained and tested for pregnancy. No IMP will be administered without first ascertaining pregnancy status. Pregnancy tests will not be done for female participants who are permanently sterile or who are post-menopausal (no menses for 12 months without an alternative medical cause).

** Blood sampling for calcium levels (including parathyroid hormone test) will only be performed at these time points if patient reports nausea and vomiting.

*** The IMP is administered once a month, every month, for 6 months.

Note: The 9-month safety check and 12-month assessments will only be performed on those patients who have received more than 6 monthly doses of the IMP prior to the protocol change dated (02.06.17). However, the IMP/placebo will no longer be administered after 6 months.

C) REFERENCE LIST

1. EMEA, *ICH Topic E 9 Statistical Principles for Clinical Trials*, 1998.
2. Moher, D., et al., *CONSORT 2010 Explanation and Elaboration: Updated guidelines for reporting parallel group randomised trials.* Journal of clinical epidemiology. **63**(8): p. e1-37.
3. White, I.R., P. Royston, and A.M. Wood, *Multiple imputation using chained equations: Issues and guidance for practice.* Statistics in Medicine, 2011. **30**(4): p. 377-399.
4. White, I.R., et al., *Strategy for intention to treat analysis in randomised trials with missing outcome data.* BMJ (Clinical research ed.), 2011. **342**: p. d40.
5. Assmann, S.F., et al., *Subgroup analysis and other (mis)uses of baseline data in clinical trials.* Lancet, 2000. **355**(9209): p. 1064-1069.
6. MacKinnon, Introduction to Statistical Mediation Analysis, 2008
7. White, I.R. and S.G. Thompson, *Adjusting for partially missing baseline measurements in randomized trials.* Statistics in Medicine, 2005. **24**(7): p. 993-1007.
8. Rothman, K.J., *No adjustments are needed for multiple comparisons.* Epidemiology (Cambridge, Mass.), 1990. **1**(1): p. 43-6.

D) AMENDMENTS

Version 1.1:

- Primary analysis changed from participants with suboptimal vitamin D at baseline to all randomised participants, this subgroup analysis was made a secondary analysis
- Removal of season as randomisation stratifier

Version 1.2:

- Amended trial statistician from Jennifer Hellier to Dominic Stringer
- Minor changes to wording

Version 1.3:

- Clarification on subpopulation (baseline vitamin D insufficient and sufficient) analysis for secondary outcomes
- Moved cardiovascular risk markers from exploratory to secondary analysis

Version 1.4:

- Further clarification of suboptimal vitamin D at baseline subgroup analysis
- Addition of sensitivity analysis to assess impact of changing inclusion criteria to allow participants taking up to 400 IUD of Vitamin D at baseline to participate in the trial

Version 1.5:

- Removal of 12 month follow up assessment and outcomes and other changes in line with protocol changes to reduce length of follow up and dosing to 6 months
- Completion of analysis of non-compliance section which had previously been left blank

Version 1.6:

- Changed sensitivity analysis to make more general to cover all instances where inclusion/exclusion criteria have changed or might change during the trial.
- Clarified blinding in respect to the analysis and to the DMC.

Version 1.7:

- Clarified secondary outcomes of PANSS subscores and Cardiovascular risk markers by listing each of separate secondary endpoints of which these consist.
- Amended some wording in line with protocol publication changes
- Added that Blood sampling vitamin D concentrations secondary outcome will be analysed last as strong expectation of a difference could lead to unblinding.
- Amended Trial Manager from Poonam Sood to Gabriella Wojewodka
- Amended methods for mediation section
- Other minor changes to wording

Version 1.8:

- Minor changes to wording/grammar corrections

Version 1.9:

- Amended CONSORT diagram to clarify wording and to add numbers for completion of outcomes at 3 and 6 month assessments
- Clarified will use time x treatment interaction in primary analysis model to allow treatment effect to vary with time
- Amended study timelines in line with extension to December 2019

Version 1.10

- Amended to clarify medians and upper/lower quartiles will be presented for descriptives as appropriate
- Minor wording changes
