# Supplementary material for: Effect of Vitamin D Supplementation on Outcomes in People With Early Psychosis: The DFEND Randomized Clinical Trial
Source: JAMA Netw Open. 2021 Dec 28;4(12):e2140858. doi: 10.1001/jamanetworkopen.2021.40858 (PMC8715346; doi:10.1001/jamanetworkopen.2021.40858)
Supplement: Supplement 2. — eMethods. Supplemental Methods eTable 1. Outcome Measures and Timeline of the DFEND Study eAppendix. Supplemental Results eTable 2. Subgroup Analysis for Efficacy Measures (PANSS), at 3- and 6-Months, With Insufficient Vitamin D at Baseline (<50 25-D Nmol/L, N = 106) eTable 3. Subgroup Analysis for Efficacy Measures (GAF, CDS, BMI, Waist Circumference, Biochemical), at 6-Months With Insufficient Vitamin D at Baseline (<50 25-D nmol/L, N = 106) eTable 4. Results From Sensitivity to Inclusion/Exclusion Criteria Analysis eTable 5. Number of Participants That Had Inpatient Stays by Trial Arm (All) eTable 6. Summary of Inpatient Days for Participants Who Had Inpatient Stays by Trial Arm (All) eTable 7. Number of Participants That Had Inpatient Stays by Trial Arm (Post-Randomisation Admissions Only) eTable 8. Summary of Inpatient Days for Participants Who Had Inpatient Stays by Trial Arm (Postrandomisation Admissions Only) eTable 9. Number of Participants That Had at Least 1 Home Treatment Contact by Trial Arm eTable 10. Summary of Home Treatment Contacts for Participants Who Had at Least 1 Home Treatment Contact by Trial Arm eTable 11. Summary of Home Treatment Contacts for All Participants by Trial Arm eTable 12. Adverse Events by Trial Arm eTable 13. Total Number of Adverse Events by Body System Code and Trial Arm eTable 14. Number of Adverse Drug Reactions by Trial Arm eTable 15. List of Adverse Drug Reactions eTable 16. Number of Serious Adverse Events by Trial Arm eTable 17. List of All SAEs eFigure 1. Sensitivity to Missing at Random Assumption eFigure 2. Mediation Diagram [file jamanetwopen-e2140858-s002.pdf]

Supplementary Online Content

Gaughran F, Stringer D, Wojewodka G, et al. Effect of vitamin D supplementation on outcomes in people with early psychosis: the DFEND randomized clinical trial. *JAMA Netw Open*. 2021;4(12):e2140858. doi:10.1001/jamanetworkopen.2021.40858

eMethods. Supplemental Methods

eTable 1. Outcome Measures and Timeline of the DFEND Study

eAppendix. Supplemental Results

eTable 2. Subgroup Analysis for Efficacy Measures (PANSS), at 3- and 6-Months, With Insufficient Vitamin D at Baseline (<50 25-D Nmol/L, N=106)

eTable 3. Subgroup analysis for Efficacy Measures (GAF, CDS, BMI, Waist Circumference, Biochemical), at 6-Months With Insufficient Vitamin D at Baseline (<50 25-D nmol/L, N=106)

eTable 4. Results From Sensitivity to Inclusion/Exclusion Criteria Analysis

eTable 5. Number of Participants That Had Inpatient Stays by Trial Arm (All)

eTable 6. Summary of Inpatient Days for Participants Who Had Inpatient Stays by Trial Arm (All)

eTable 7. Number of Participants That Had Inpatient Stays by Trial Arm (Post-Randomisation Admissions Only)

eTable 8. Summary of Inpatient Days for Participants Who Had Inpatient Stays by Trial Arm (Postrandomisation Admissions Only)

eTable 9. Number of Participants That Had at Least 1 Home Treatment Contact by Trial Arm

eTable 10. Summary of Home Treatment Contacts for Participants Who Had at Least 1 Home Treatment Contact by Trial Arm

eTable 11. Summary of Home Treatment Contacts for All Participants by Trial Arm

eTable 12. Adverse Events by Trial Arm

eTable 13. Total Number of Adverse Events by Body System Code and Trial Arm

eTable 14. Number of Adverse Drug Reactions by Trial Arm

eTable 15. List of Adverse Drug Reactions

eTable 16. Number of Serious Adverse Events by Trial Arm

eTable 17. List of All SAEs

eFigure 1. Sensitivity to Missing at Random Assumption

eFigure 2. Mediation Diagram

This supplementary material has been provided by the authors to give readers additional information about their work.

© 2021 Gaughran F et al. *JAMA Network Open.*

eMethods.

Vitamin D analysis

Vitamin D was assessed by LC/MS in serum samples taken at baseline and Month 6 of the study (Viapath, Nutristasis Unit, London, UK, DEQAS certified). 25(OH)D3/D2 analysis in serum/plasma was performed using an automated protein crash, solid phase extraction using the Gerstel Multi-Purpose Sampler (MPS). Sample analysis is fully automated by means of an external injection valve and loop fitted onto the MPS. Separation of the 25(OH)D3 and 25(OH)D2 from the sample matrix was performed using an Agilent Eclipse Plus C18 2.1 x 50mm; 3.5 µm particle size. The chromatographic mobile phases consisted of 0.1 % acetic acid (v/v) in water (eluent A) and 0.1 % acetic acid (v/v) in methanol (eluent B). A gradient elution was performed from 80 % B to 90 %B in 2 minutes, with an isocratic hold at 90% for 0.5 minutes; the column was then equilibrated to baseline conditions. Column flow rate was 0.5ml/min throughout the chromatographic run whilst the column temperature was maintained at 50°C. An Agilent 6460 tandem mass spectrometer with the multimode source was used in positive simultaneous ESI/APCI mode. The multiple-reaction monitoring (MRM) transitions used for quantification of 25(OH)D3, 25(OH)D2 and hexadeuterated 25(OH)D3 were m/z 401.3 > 383.3, 413.3 > 395.3 and 407.3 > 389.3, respectively. Hexadeuterated internal standard, 25OHD3 and 25OHD2 eluted from the column at 2.22, 2.23 and 2.32 min, respectively.

eTable 1. Outcome Measures and Timeline of the DFEND Study

**STUDY PERIOD**	**SCREENING AND BASELINE**	**TREATMENT Vigantol®/Placebo**						**Follow-up phone call**
**TIMEFRAME**		**Month 1**	**Month 2**	**Month 3**	**Month 4**	**Month 5**	**Month 6**	**28 days after last dose**
Informed consent & Eligibility	X							
Treatment of IMP or placebo		←					→	
Sociodemographics	X							
NOS (Duration of Untreated Psychosis)	X							
Concomitant medication	X	X	X	X	X	X	X	
Medical history	X							
Vitamin D supplementation check	X	X	X	X	X	X	X	
Anthropometrics	X						X	
Blood sampling: vitamin D concentration (storage)	X						X	
Blood sampling: incl. clinical, genetic, cardiovascular and inflammatory markers	X						X	
Urine pregnancy test	X*	X*	X*	X*	X*	X*	X*	
Smoking habits	X						X	
Positive and Negative Syndrome Scale (PANSS)	X			X			X	

© 2021 Gaughran F et al. *JAMA Network Open.*

Global Assessment of Functioning (GAF)	X						X	
Calgary Depression Scale (CDS)	X						X	
OPCRIT	X							
Sun Exposure questionnaire	X						X	
International Physical Activity Questionnaires (IPAQ)	X						X	
Simple Physical Activity Questionnaire (SIMPAQ)	X						X	
Fitzpatrick Skin Type Questionnaire	X							
Vitamin D Food Frequency Questionnaire	X						X	
Adverse events (AEs)**	X	X	X	X	X	X	X	X
Blood sampling for calcium levels	X	X***	X***	X	X***	X***	X	
Patient Trial Medication Guess		X	X	X	X	X	X	X
Service Contacts Form	X						X	

* In the event that it was not possible to obtain a urine sample, a blood HCG sample was obtained for pregnancy testing. No IMP was administered without first ascertaining pregnancy status. Pregnancy tests were not done for female participants who were permanently sterile or who were post-menopausal (no menses for 12 months without an alternative medical cause).
** AEs were collected from consent. Patients were contacted 28 days after the last dose of treatment to check for any AEs. At dosing visits 1 – 6, AEs were collected in person.
*** Blood sampling for calcium levels (including parathyroid hormone test) was only performed at these time points if patient reported nausea and vomiting.

Supplemental Statistical Methods

The multiple imputation procedure involved first identifying predictors of missingness of PANSS at 6 months. This was done using stepwise (backwards-selection) logistic regression and the following variables were considered: trial arm, age, sex, ethnicity, site, baseline BMI, baseline smoking status, baseline PANSS, baseline CDS score, baseline GAF disability score, baseline GAF symptoms score and compliance to the intervention (parameterised as number of doses taken of the intervention or placebo). These variables covered the main demographic variables (defined in the SAP and described in this report) and all baseline values of the outcome variables that also had few or no missing values.

In this stepwise model, all variables were included and, in each step, the variable with the largest p-value was removed from the model until all remaining variables predicted missingness at a liberal significance level of p<0.10. In this procedure, age and compliance were found to be predictors of missingness. Multiple imputation was performed using the mi command in Stata 15.1 with sequential imputation using chained equations (MICE). [1] We constructed an imputation model for each primary and secondary outcome to impute missing values in the respective response and explanatory variables. These imputation models included all the variables from the respective analysis model (including trial arm, ethnicity vitamin D insufficiency and interaction between trial arm and vitamin D insufficiency [for subgroup analyses] and baseline value of outcome) as well as age and compliance (number of doses taken). 100 imputations were used for each outcome.

Baseline vitamin D insufficiency and an interaction between trial arm and vitamin D insufficiency were

© 2021 Gaughran F et al. *JAMA Network Open.*

included in the imputation models to allow treatment effects to vary with vitamin D insufficiency status for the estimation of the baseline vitamin D insufficient subpopulation treatment effect. These terms had to be imputed themselves as there were 7 missing values of vitamin D insufficiency status at baseline. The interaction term was included as “just another variable” in the imputation models rather than insisting on consistency between imputed values for the interaction term and the vitamin D insufficiency variable. This has been shown to be a reasonable approach when there are only a few missing values.[2] Values for baseline vitamin D insufficiency and the interaction were imputed using predictive mean matching with k=10 nearest neighbours as using logistic regression models to impute both variables led to perfect prediction and models did not converge.

For consistency we used the same set of imputation models but different analysis models to estimate treatment effects in the original target population and to estimate treatment effects in the subpopulation of vitamin D deficient patients. We first describe the former, and then explain how these analysis models were extended to estimate treatment effects in the subpopulation of deficient trial participants.

To estimate treatment effects in the whole target population we used separate imputation models for each outcome except for PANSS total and PANSS subscores at 3 and 6 months which were related variables and for the two GAF scores at 6 months which are also related concepts. For PANSS a single imputation model was used to impute the six subscores at 3 and 6 months, and then the PANSS total was derived from these scores. This imputation model included baseline PANSS total as well as baseline PANSS subscores as predictors in addition to the above. Similarly, a single imputation model was used to impute the two GAF scores (Symptoms and Disability) with both baseline scores (Symptoms and Disability) included as predictors in the model.

For each outcome, the analysis model used was a linear regression with treatment arm, baseline outcome and ethnicity (randomisation stratifier) as covariates. As mentioned above, the imputation models contained all the variables of the analysis model(s) as well as missingness predictors age and compliance. All the variables that needed imputing were continuous variables, principally the outcome variables and baseline covariates for some of the outcomes. To impute values for these variables, regression models were used unless they produced unrealistic imputations. When using linear regression as the imputation model, a substantial number of imputed values for PANSS subscores tended to be outside the range of valid values, and the same issue arose for the CDS and vitamin D variables. Therefore, these variables were imputed using the alternative approach “predictive mean matching” with imputed values drawn from the 10 “nearest neighbours” (as recommended by Morris, et al.,2014)[3] to ensure that imputed values lay within the permissible range. Finally, analysis models were run for each imputed dataset and estimates combined using Rubin’s rules (as performed by the mi estimate command).

For the vitamin D subgroup analyses, the analysis model was expanded to include baseline vitamin D status and the interaction product term as extra explanatory variables; with the chosen coding ensuring that the regression coefficient of trial arm represented the treatment effect within the subpopulation of vitamin D insufficient FEP patients.

For the month 6 vitamin D blood concentration outcome, the imputation model did not include baseline vitamin D insufficiency or the interaction as the imputation model already included baseline vitamin D blood concentration as it features as a covariate in the analysis model. An exploratory analysis (not specified in the SAP) was added for the vitamin D insufficient subgroup for this outcome; the imputation model for this analysis featured baseline vitamin D insufficiency and the interaction with trial arm, but not baseline vitamin D blood concentration. The analysis model for this analysis

used baseline vitamin D insufficiency (and the interaction) as covariates and not baseline vitamin D blood concentration.

Two sensitivity analyses were carried out for the primary outcome. Firstly, sensitivity to changes to the inclusion/exclusion criteria. The inclusion/exclusion criteria changed 3 times throughout the trial and so the population being sampled changed. Each change was a relaxation of the criteria. For these sensitivity analyses, the same analysis model as for the primary outcome was run but for restricted samples. The sensitivity analyses used data imputed on the whole sample (the same imputed data as used for per primary analysis) and then the sensitivity analyses were run on the relevant subsamples. The subsamples correspond to each of the inclusion/exclusion criteria changes.

The second sensitivity analysis is to check the robustness of the results against departures from the missing at random assumption for the primary analysis. In the main primary analysis, we have assumed that the missing data is missing at random; chiefly that missingness of values of PANSS total at Month 6 only depends on data collected (specifically the variables used in the multiple imputation model). For this sensitivity analysis, we used the rctmiss package in Stata 15 to carry out the primary analysis under a range of assumptions about the missing data, where the data and missingness are modelled jointly using a pattern-mixture model. The same regression model was used as in the primary analysis, and age and number of doses taken defined as auxiliary variables as they also featured in the multiple imputation model for the primary analysis. Vitamin D insufficiency variables were not included for simplicity. The scenarios show the estimated treatment effect (with confidence intervals) for different values of delta, where delta is defined as the difference in the mean of the unobserved values of the outcome from the mean of the adjusted observed values (in specified arms). We have chosen values of delta from -15 to +15 on the PANSS total score, based on 15 being approximately 1 standard deviation in PANSS total score observed at baseline (and so representing a large difference between the mean of the unobserved and observed values).

Finally, a mediation analysis was carried out to test the hypothesis that vitamin D blood levels are a mediator of the effect of treatment on PANSS total score. A complete case (participants with complete data on the variables in the model) regression-type mediation analysis was carried out (using the paramed command in Stata 15 to allow for an interaction between treatment and the mediator) with PANSS total score at Month 6 as the outcome, Month 6 vitamin D blood concentration levels (mmol/L) as the mediator and randomisation arm (Vitamin D or Placebo) as the treatment variable. Linear regression models were fit for the outcome and the mediator. Baseline PANSS score and Ethnicity were included as covariates in all regression models as per the primary analysis model, as well as baseline vitamin D blood concentration levels (mmol/L).

1. Raghunathan TE, Lepkowski J, Hoewyk JV, Solenberger P. A multivariate technique for multiply imputing
missing values using a sequence of regression models. Survey Methodology 2001; 27: 85-95.
2. Seaman SR, Bartlett JW, White IR. Multiple imputation of missing covariates with non-linear effects and
interactions: an evaluation of statistical methods. BMC Med Res Methodol 2012; 12: 46.
3. Morris TP, White IR, Royston P. Tuning multiple imputation by predictive mean matching and local residual draws. BMC Medical Research Methodology 2014; 14(1): 75.

© 2021 Gaughran F et al. *JAMA Network Open.*

eAppendix. Supplemental Results

Three changes occurred to the inclusion/exclusion criteria throughout recruitment to the trial; these were all relaxations of the inclusion/exclusion criteria such that a greater number of potential participants were eligible. Below we have estimated the average treatment effect for the primary outcome (PANSS) in the subpopulations reflected by these changes. As above, this uses data imputed on the whole sample (the same imputed data as used for per primary analysis) and then the sensitivity analyses are run on the relevant subsamples. This ensures that any differences between these results and the primary outcome are not due to different imputation models.Table S4 shows the treatment effect estimates after increasingly widening the target population. This shows that while the estimated group difference varied somewhat in size, they are consistently in the same direction and not statistically significant. Thus, our finding is robust in terms of choice of eligibility criteria.

eFigure shows scenarios based on potential values of PANSS total at month 6 for the missing observations were the missing at random assumption were false. The scenarios are what the estimated treatment effect (with confidence intervals) would be for different values of delta, where delta is the difference in the mean of the unobserved values of the outcome from the mean of the adjusted observed values (in specified arms). The y axis gives the estimated PANSS difference with limits we would have estimated by the primary analysis model under these different scenarios (values of delta). We have chosen values of delta from -15 to +15 on the PANSS total score, based on this being approximately 1 standard deviation in PANSS total score observed at baseline (and so representing a large difference).

For the primary analysis (PANSS at month 6), the estimated treatment effect was in favour of the Placebo arm (as the treatment effect was positive, and higher PANSS scores are worse). Therefore, the question of interest is "What would the missing data need to look like for there to be evidence of a treatment effect in the other direction?" (The hypothesised treatment effect was that Vitamin D was neuroprotective for first-episode psychosis patients.) This could occur if the unobserved PANSS scores were lower than the observed scores in the Vitamin D arm, or more plausibly if the unobserved PANSS scores were higher than the observed scores in the Placebo arm.

The sections of the blue and red lines below zero in the Figure above correspond to these two scenarios. From the blue line, we can see that even if the mean PANSS scores in the unobserved data for the Vitamin D arm were 15 points lower than the mean observed PANSS scores, the treatment effect would be in the hypothesised direction but there still would not have been evidence ($p<0.05$) of a treatment effect as the confidence interval includes zero.

Similarly, if the missing PANSS scores in the Placebo arm were on average 15 points higher than the observed PANSS scores, there would not have been evidence ($p<0.05$) of a treatment effect.

In conclusion, even if the departure from the missing at random assumption was substantial (15 PANSS points difference in either arm), there would still not be evidence of a treatment effect in favour of Vitamin D.

A straightforward (complete case) mediation was carried out to check if vitamin D blood levels were a mediator of Total PANSS score

Number of inpatient days were calculated from the Service Contacts form recorded at baseline and at 6 months, by summing the number of inpatient days post-randomisation for inpatient stays recorded on the form. Where the end date for the inpatient stay was missing, this was assumed to be the last contact with the participant (withdrawal or the last visit they attended up to Month 6). For inpatient stays that started pre-randomisation, start date was taken as date of randomisation for the purpose of calculating inpatient days post-randomisation.

© 2021 Gaughran F et al. *JAMA Network Open.*

Of those randomised to vitamin D, 18.9% had an inpatient stay over the course of their inclusion in the trial, with the comparable figure in control patients being 25.3%. Mean(SD) and median(LQ-UQ) length of inpatient stays in those admitted were 33.5(31.1), 23.5(8-48) in the vitamin D group and 27.8(31.9), 15(6-49) in the control group.

Number of home treatment team contacts was calculated as the sum of all home treatment team contacts at home, at service base/other service setting by telephone and by unspecified location as recorded on the Service Contacts form.
In the vitamin D Group 5% (SD6.8%) had at least one home treatment contact, with the comparable figure in the placebo group being 7(SD9.3%). Of those in contact with Home treatment services, the vitamin D group had a mean of 15.4% (5.4) contacts and the control group (12.6 (6.3)

Overall, 66 patients (44%) patients experienced at least one AE with a total of 119 AEs reported. The proportion of all trial participants that experienced at least one SAE was 12% (n=18). Safety results for the DFEND study are summarised in the tables below.

A total of 6/119 AEs (5%) were assessed as related to at least one study drug and 5/149 patients (3%) experienced an ADR. There were no Serious Adverse Reactions (SARs), no unexpected SARs and no SUSARs.

eTable 2. Subgroup Analysis for Efficacy Measures (PANSS), at 3- and 6-Months, With Insufficient Vitamin D at Baseline (<50 25-D nmol/L, N=106)

	3-month		6-month	
Outcome measures	Mean difference, VitD-Placebo (95% CI)	*p*-value	Mean difference, VitD-Placebo (95% CI)	*p*-value
PANSS total score	-2.57 (-7.93, 2.79)	.34	3.21(-2.21, 8.63)	.24
PANSS Positive symptoms score	-1.10 (-2.55, 0.35)	.14	0.37 (-1.20, 1.94)	.64
PANSS Negative symptoms score	0.92 (-1.48, 3.31)	.45	1.63 (-0.54, 3.81)	.14
PANSS General Psychopathology score	-2.37 (-5.06, 0.32)	.08	1.19 (-1.99, 4.39)	.46

PANSS: Positive and Negative Symptoms Scale

eTable 3. Subgroup analysis for Efficacy Measures (GAF, CDS, BMI, Waist Circumference, Biochemical), at 6-Months With Insufficient Vitamin D at Baseline (<50 25-D nmol/L, N=106)

Outcome measures (6-months)	Mean difference, VitD-Placebo (95% CI)	*p*-value

GAF Symptom score	0.45(-5.34, 6.24)	.88
GAF Disability score	1.13 (-5.05, 7.32)	.72
CDS score	-0.55 (-2.52, 1.41)	.58
Waist Circumference, cm	-2.13 (-6.34, 2.09)	.32
BMI, kg/m^2	0.01 (-1.08, 1.10)	.96
HbA1c, mmol/mol	-0.58 (-2.33, 1.17)	.51
Total Cholesterol, mmol/L	0.07 (-0.42, 0.55)	.78
C-Reactive Protein (>3), mg/L	0.28 (0.05, 1.56)*	.15

*Odds ratio

GAF: Global Assessment of Functioning; CDS: Calgary Depression Scale; BMI: Body Mass Index

eTable 4. Results From Sensitivity to Inclusion/Exclusion Criteria Analysis

Population	N	Mean Difference	SE	Lower 95% CI	Upper 95% CI	p-value
**i)**	29	3.357	5.319	-7.873	14.587	0.536
**ii)**	62	1.226	3.932	-6.708	9.160	0.757
**iii)**	74	2.231	3.673	-5.137	9.599	0.546
**iv)**	149	3.570	2.362	-1.110	8.250	0.134

i) Including all participants recruited under Version 3 24.08.2015, Version 4 11.04.2016 or Version 5.1 07.07.16* (no I/E changes) of the DFEND trial protocol. See appendix 1 for full inclusion/exclusion criteria as of Version 4.

ii) Including all participants recruited under Version 6 02.12.16 of the DFEND trial protocol or previous versions as per ii). I/E in Version 6 was amended such that the exclusion criteria “Those who are currently taking vitamin D supplements” became “Those who are currently taking vitamin D supplements at a dose exceeding 400IU/day” and the inclusion criteria “Willing to agree to refrain from taking multivitamin or non-study vitamin D supplements throughout the study” became “Willing to agree to refrain from taking multivitamin or non-study vitamin D supplements that exceed 400IU/day throughout the study”.

iii) Including all participants recruited under Version 7 02.06.17 of the DFEND trial protocol or previous versions as per iii). I/E in Version 7 was amended such that the inclusion criteria “Aged between 18-45 years old including women of child-bearing age” became “Aged between 18- 65 years old including women of child-bearing age”.

iv) Including all recruited participants. The inclusion/exclusion criteria was amended in Version 8 23.08.17 to remove the exclusion criteria “Known current anaemia, sickle cell anaemia and beta or alpha thalassemia”. Further revisions were made to the protocol, but no further inclusion/exclusion criteria changes were made, and revisions did not affect the target study population. Current version of the protocol is V9.1 06.06.2018.

eTable 5. Number of Participants That Had Inpatient Stays by Trial Arm (All)

Inpatient stay	Placebo	Vitamin D	Total
No	56 (74.7%)	60 (81.1%)	116 (77.9%)
Yes	19 (25.3%)	14 (18.9%)	33 (22.2%)

These summary statistics excludes all patients with zero days.

eTable 6. Summary of Inpatient Days for Participants Who Had Inpatient Stays by Trial Arm (All)

Trial Arm	N	Mean (SD)	Median (LQ-UQ)	Range
Placebo	19	27.84 (31.93)	15 (6 – 49)	1 - 125
Vitamin D	14	33.50 (31.09)	23.5 (8 – 48)	2 - 94
Total	33	30.24 (31.21)	17 (7 – 48)	1 - 125

Number of inpatient days here were calculated in the same way as in Section 1, except any stays that started pre-randomisation were excluded and counted as 0 days.

eTable 7. Number of Participants That Had Inpatient Stays by Trial Arm (Post-Randomisation Admissions Only)

Inpatient stay	Placebo	Vitamin D	Total
No	63 (84.0%)	70 (94.6%)	133 (89.3%)
Yes	12 (16.0%)	4 (5.4%)	16 (10.7%)

eTable 8. Summary of Inpatient Days for Participants Who Had Inpatient Stays by Trial Arm (Post-Randomisation Admissions Only)

Trial Arm	N	Mean (SD)	Median (LQ-UQ)	Range
Placebo	12	24.00 (20.69)	15 (10 – 39.5)	2 - 64
Vitamin D	4	32.00 (41.27)	17 (5 – 59)	2 - 92
Total	16	26.00 (25.84)	15 (7 – 39.5)	2 - 92

eTable 9. Number of Participants That Had at Least 1 Home Treatment Contact by Trial Arm

>=1 HTT contact	Placebo	Vitamin D	Total
No	68 (90.7%)	69 (93.2%)	137 (92.0%)
Yes	7 (9.3%)	5 (6.8%)	12 (8.1%)

© 2021 Gaughran F et al. *JAMA Network Open.*

eTable 10. Summary of Home Treatment Contacts for Participants Who Had at Least 1 Home Treatment Contact by Trial Arm

Trial Arm	N	Mean (SD)	Median (LQ-UQ)	Range
Placebo	7	12.57 (6.27)	14 (8 -16)	1-20
Vitamin D	5	15.40 (5.37)	16 (16 – 16)	7-22
Total	12	13.75 (5.83)	16 (10.5 – 16)	1-22

eTable 11. Summary of Home Treatment Contacts for All Participants by Trial Arm

Trial Arm	N	Mean (SD)	Median (LQ-UQ)	Range
Placebo	75	1.17 (4.09)	0 (0-0)	0-20
Vitamin D	74	1.04 (4.09)	0 (0-0)	0-22
Total	149	1.11 (4.08)	0 (0-0)	0-22

eTable 12. Adverse Events by Trial Arm

Trial Arm	Total AEs	Number of participants	Percentage of pts	Mean number of AEs per participant
Placebo	62	37	49.3%	0.83
Vitamin D	57	29	39.2%	0.77

eTable 13. Total Number of Adverse Events by Body System Code and Trial Arm

Body System Code	Placebo	Vitamin D	Total
1. Cardiovascular	2 (50.0%)	2 (50.0%)	4 (3.4%)
2. Respiratory	4 (23.5%)	13 (76.5%)	17 (14.3%)
3. Hepatic	1 (100.0%)	0 (0.0%)	1 (0.8%)
4. Gastro-intestinal	11 (44.0%)	14 (56.0%)	25 (21.0%)
5. Genito-urinary	3 (75.0%)	1 (25.0%)	4 (3.4%)
6. Endocrine	NA (-)	2 (100.0%)	2 (1.7%)
7. Hematological	1 (100.0%)	0 (0.0%)	1 (0.8%)
8. Musculo-skeletal	5 (62.5%)	3 (37.5%)	8 (6.7%)
10. Neurological	3 (75.0%)	1 (25.0%)	4 (3.4%)
11. Psychological	21 (61.8%)	13 (38.2%)	34 (28.6%)
12. Immunological	1 (100.0%)	0 (0.0%)	1 (0.8%)
13. Dermatological	1 (50.0%)	1 (50.0%)	2 (1.7%)

© 2021 Gaughran F et al. *JAMA Network Open.*

Body System Code	Placebo	Vitamin D	Total
15. Eyes, ear, nose, throat	2 (100.0%)	0 (0.0%)	2 (1.7%)
17. Other (please specify)	7 (50.0%)	7 (50.0%)	14 (11.8%)

eTable 14. Number of Adverse Drug Reactions by Trial Arm

Trial Arm	Total ADRs	Number of participants	Percentage of pts	Mean number of ADRs per participant
Placebo	4	3	4.0%	0.05
Vitamin D	2	2	2.7%	0.03

eTable 15. List of Adverse Drug Reactions

Participant Number	ADR Description	Trial Arm	Related to study treatment?
1	DIARRHOEA AFTER LAST DOSE	Vitamin D	3. Possible
2	RAW STOMACH ACHE 1 HOUR AFTER DOSE LASTING FOR 1 HOUR	Placebo	3. Possible
3	WORSENING OF PERSECUTORY DELUSIONS (ADMITTED AS AN INPATIENT)	Placebo	3. Possible
3	WORSENING OF PERSECUTORY DELUSIONS - HTT SUPPORT REQUIRED	Placebo	3. Possible
4	NAUSEA	Vitamin D	3. Possible
5	NAUSEA	Placebo	3. Possible

Note: "Participant Number" is a dummy identifier used to show where multiple AEs occurred for the same participant.

eTable 16. Number of Serious Adverse Events by Trial Arm

Trial Arm	Total SAEs	Number of participants	Percentage of pts	Mean number of SAEs per participant
Placebo	13	12	16.0%	0.17
Vitamin D	8	6	8.0%	0.11

eTable 17. List of All SAEs

Participant Number	SAE Description	Date started	Related to Study treatment?	Trial Arm
1	PSYCHOTIC EXACERBATION	18/07/2016	5. None	Placebo
2	PSYCHOTIC EXACERBATION	28/06/2016	5. None	Placebo
3	PSYCHOTIC EXACERBATION	26/12/2016	5. None	Vitamin D

© 2021 Gaughran F et al. *JAMA Network Open.*

Participant Number	SAE Description	Date started	Related to Study treatment?	Trial Arm
4	PSYCHOTIC EXACERBATION (HOSPITALISATION)	26/09/2016	5. None	Placebo
4	PSYCHOTIC EXACERBATION (HOSPITALISATION)	04/11/2016	5. None	Placebo
5	DETERIORATION IN MENTAL HEALTH	26/09/2016	5. None	Placebo
6	PSYCHOTIC EXACERBATION	14/02/2017	5. None	Placebo
7	PSYCHOTIC EXACERBATION	21/02/2017	5. None	Placebo
8	PSYCHOTIC EXACERBATION LEADING TO HOSPITAL ADMISSION.	03/10/2017	5. None	Placebo
9	PSYCHOTIC RELAPSE - INPATIENT ADMISSION	16/02/2018	5. None	Placebo
10	WORSENING OF PERSECUTORY DELUSIONS (ADMITTED AS AN INPATIENT)	05/07/2018	3. Possible	Placebo
11	DETERIORATION IN MENTAL STATE LEADING TO HOSPITAL ADMISSION	09/04/2019	4. Remote	Vitamin D
12	OVERDOSE	22/03/2017	5. None	Placebo
13	DEPRESSIVE EPISODE WITH FEATURES OF PSYCHOSIS	31/08/2017	5. None	Vitamin D
14	ATTEMPTED SUICIDE/SELF-HARM	05/12/2017	5. None	Placebo
15	ABDOMINAL PAIN DUE TO ACID REFLUX	04/06/2018	5. None	Vitamin D
16	OVERDOSE	21/08/2017	5. None	Placebo
17	VISUAL HALLUCINATIONS, PANIC - DIAGNOSED POSSIBLE RELAPSE	23/03/2018	5. None	Vitamin D
17	PANIC	30/03/2018	5. None	Vitamin D
18	OLFACTORY HALLUCINATION - SMELLS AIR FRESHENER ON STREETS	01/07/2018	4. Remote	Vitamin D
18	PSYCHOTIC EPISODE	03/09/2018	4. Remote	Vitamin D

Note: “Participant Number” is a dummy identifier used to show where multiple AEs occurred for the same participant.

	25(OH)D3	25(OH)D2
inter-assay (CV%)	10	10
intra-assay (CV%)	5.0	6.3

© 2021 Gaughran F et al. *JAMA Network Open.*

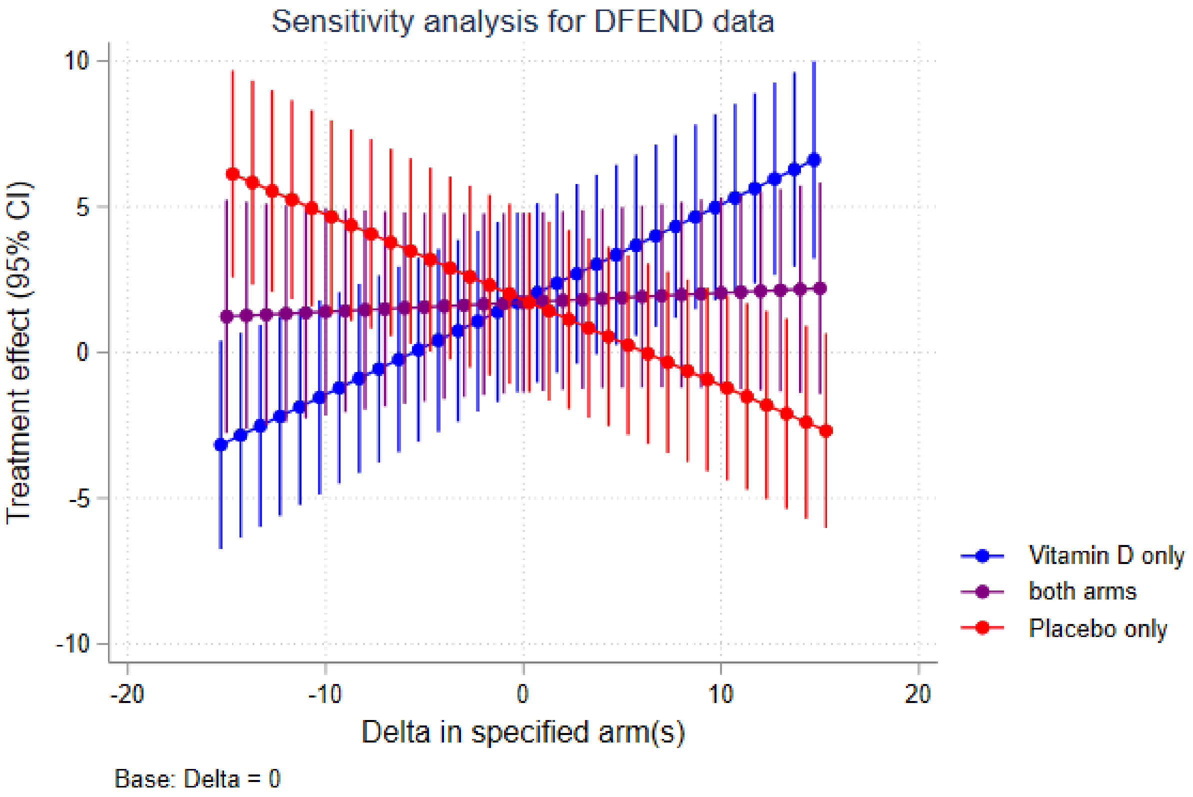

eFigure 1. Sensitivity to Missing at Random Assumption

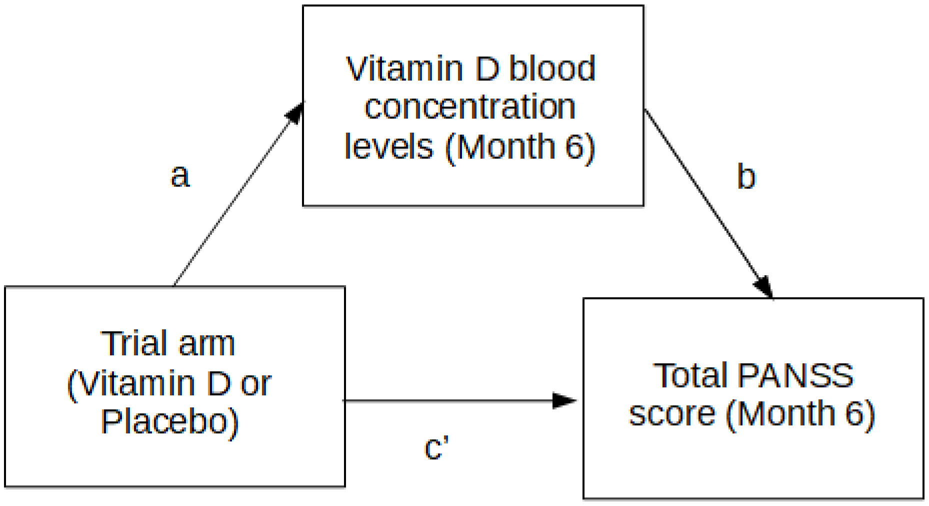

eFigure 2. Mediation Diagram

The estimate of the **a** path was 41.64 (CI from 29.13 to 54.14, p<0.001) in line with the secondary outcome results for Vit D. The estimate of the **b** path was -0.21 (-0.58,0.16, p=0.271). The natural direct effect (**c'**) was 0.463 (95% CI --8.01, 7.37, p=0.899) and the natural indirect effect was 2.50 (-3.81, 9.76, p=0.275). The marginal total effect (corresponding

© 2021 Gaughran F et al. *JAMA Network Open.*

to the primary analysis) was 2.96 (--2.33,8.48, p=0.289). Whilst there was clearly evidence of an effect of trial arm on vitamin D blood levels, there was no evidence for a mechanism whereby blood vitamin D levels were affecting PANSS and hence there was no evidence of changes in PANSS being brought about by changes in blood vitamin D blood levels (i.e. no mediation), although it is pertinent to note that power was lacking to detect a mediation effect.

© 2021 Gaughran F et al. *JAMA Network Open.*
