# Supplementary material for: Effect of Vitamin D Supplementation on Outcomes in People With Early Psychosis: The DFEND Randomized Clinical Trial
Source: JAMA Netw Open. 2021 Dec 28;4(12):e2140858. doi: 10.1001/jamanetworkopen.2021.40858 (PMC8715346; doi:10.1001/jamanetworkopen.2021.40858)
Supplement: Supplement 3. — Data Sharing Statement [file jamanetwopen-e2140858-s003.pdf]

Data Sharing Statement

Gaughran. Effect of Vitamin D Supplementation on Outcomes in People With Early Psychosis. *JAMA Netw Open*. Published December 28, 2021. doi:10.1001/jamanetworkopen.2021.40858

Data
Data available: Yes
Data types: Deidentified participant data
How to access data: Supplementary data included in files
When available: With publication

Supporting Documents
Document types: None

Additional Information
Who can access the data: researchers whose proposed use of the data has been approved
Types of analyses: Analyses in keeping with those permitted
Mechanisms of data availability: with a signed data access agreement
Any additional restrictions: Data is anonymised
